# European blackflies: accepted and questionable species (Diptera, Simuliidae)

**DOI:** 10.3897/zookeys.1291.196803

**Published:** 2026-09-14

**Authors:** Ladislav Jedlička, Matúš Kúdela, Tatiana Kúdelová

**Affiliations:** 1 Department of Zoology, Comenius University, Ilkovičova 6, SK-84215 Bratislava, Slovakia Department of Zoology, Comenius University Bratislava Slovakia

**Keywords:** Accepted species, blackflies, Europe, questionable species, species status

## Abstract

Primary and secondary published sources on European blackflies were systematically examined, and relevant data were extracted and compiled into a comprehensive inventory of extant species. All non-synonymised species-level taxa reported from Europe were incorporated into a raw data pool, comprising 268 taxa. A basic core set of species, containing 261 taxa, was derived from the raw data pool by excluding formally unnamed taxa—specifically two morphoforms and five cytoforms (including one hypothetical species)—and two *nomina nuda*. This also included the reassessment of *Simulium aff. tuberosum* and the revalidation of two species: *Simulium annulipes* Becker, 1908 (cytoform C of *Simulium ruficorne* Macquart, 1838) and *Simulium beckeri* Roubaud, 1906 (cytoforms A1/A2 of *S. ruficorne*). The core set of species was subsequently subdivided into two principal subsets: the List of accepted species, comprising 233 taxa valid under the ICZN and reliably documented in Europe, and the List of questionable species, comprising 28 taxa requiring further verification due to unresolved taxonomy, uncertain identifications, or insufficient evidence of their occurrence in Europe.

## Introduction

A species list, or checklist, is a taxonomically organised inventory of species of a particular group documented within a defined geographic area and time period (Mayr 1969; Thompson and Knutson 1987; Mayr and Ashlock 1991; Reyserhove et al. 2020). Depending on their intended purpose, checklists may range from simple lists of valid names, through nomenclatural and taxonomic checklists including references to nomenclatural acts and taxonomic treatments, to annotated checklists enriched with comments or supplementary data, and ultimately to comprehensive species accounts providing information on nomenclature, taxonomy, diagnostic characters, bibliographic sources, and distribution (Thompson and Knutson 1987; GBIF 2017).

Such checklists provide authoritative baselines and constitute essential tools across a wide spectrum of research and decision-making processes. Their principal value lies in offering authoritative foundation of species nomenclature, taxonomy, and distribution which underpin advanced analyses in biogeography, macroecology, biodiversity research, parasitology and public health, conservation, and other related disciplines. In all cases, checklists serve as both a key source of information and a taxonomic framework supporting studies across disciplines, whether for research or policy (Gotelli 2004; Jones et al. 2012; Garnett et al. 2020; Reyserhove et al. 2020; Thomson et al. 2021). However, checklists are neither definitive nor static; they should be continually updated to reflect new species descriptions, taxonomic revisions, and distributional records, ensuring their continued relevance and scientific accuracy (Jones et al. 2012).

Well-structured checklists are particularly important and in high demand for groups of organisms with substantial practical relevance, especially those that affect human activities and health. Blackflies (Diptera: Simuliidae) represent one such group, having attracted scientific attention since the earliest zoological studies and even earlier, owing to their role as some of the most significant blood-sucking arthropods (Crosskey 1990). The females of most species feed on the blood of mammals and birds, and their bites can provoke severe reactions. Blood-feeding is also associated with the capacity to transmit various diseases, most notably human onchocerciasis (river blindness) in tropical Africa, South America, and Yemen (Crosskey 1990; Werner and Grunewald 2010; Adler and McCreadie 2019; WHO 2021).

In Europe, blackflies are chiefly recognised as haematophagous parasites of mammals, including humans, and of birds. When describing the first two blackfly species—*Culex reptans* and *C. equinus*—Linnaeus (1758) already noted their blood-sucking habits and the nuisance they pose to humans, with even more vivid accounts provided earlier in Fauna Suecica (Linnaeus 1746: 328–329). Although European blackflies are not known to transmit major human parasites, they are recognised vectors of avian haemosporidian parasites (Chakarov et al. 2020; Žiegytė and Bernotienė 2022), and potential vectors of pathogenic bacteria (*Bartonella* spp., Šikutová et al. 2026) and zoonotic filarial nematodes (Otranto et al. 2012, 2013). They are, however, best known for their detrimental effects on livestock, including documented cases of cattle mortality (e.g. Wilhelmi 1920; Werner and Grunewald 2010).

Historically, the most prominent European species was *Simulium colombaschense* (Scopoli), which bred in enormous numbers in the middle reaches of the Danube River, particularly in the Iron Gates gorge (Živković 1975; Crosskey 1990). Its populations collapsed following the construction of the Iron Gates Dams in the 1970s, and the species has not been detected in this section of the Danube recently (Živković 1975; Adler et al. 2016a). Seasonally high biting activity or sporadic outbreaks of several blackfly species have nevertheless been reported from various parts of Europe (e.g. Wilhelmi 1920; Bel’tyukova 1962, 1977; Bel’tyukova and Leykina 1964; Jedlička and Halgoš 1982; Betke 2003; Malmqvist et al. 2004; Bartninkaité et al. 2006; Ignjatović Ćupina et al. 2006, 2014; Kaplich et al. 2008; Werner and Kampen 2009; Ruiz-Arrondo et al. 2020; López Peña et al. 2021; Sitarz et al. 2022). Daily biting rates commonly ranged from 10 to > 1,000 on cattle, with even higher values reported for *S. colombaschense* (Živković 1975). Annual biting rates documented for European blackflies ranged from 15,000 to 50,000 per year on horses (Jedlička and Halgoš 1982).

A second major aspect of the significance of black flies lies in their role within aquatic trophic networks of lotic ecosystems, particularly in boreal regions (Malmqvist et al. 2004; Adler and Courtney 2019). Despite its importance, this role remains largely unrecognised by the public and is often underestimated even within hydrobiological research (Boóz et al. 2024).

Blackflies are therefore not solely the concern of specialist taxonomists; they are relevant to a wide range of disciplines, including biodiversity assessment, hydrobiology, faunistics, zoogeography, public health, environmental management, and related fields. In all these domains, research depends on a robust taxonomic framework and accurate knowledge of species composition in the area of interest—the primary output of which is a reliable species checklist.

The first modern and comprehensive checklist of Pan-European simuliids was published within the Fauna Europaea framework (Crosskey 2004, 2013, 2017). Although the Fauna Europaea platform is currently inaccessible, a cartographic derivative remains available via PESI (De Jong et al. 2015; PESI 2024); we have archived a copy of the originally published dataset in our institutional repository. Subsequent Fauna Europea editions largely reproduced the 2004 dataset with only minor revisions and are therefore now partially outdated.

The most current and complete global list of blackfly species is maintained in the annually revised Inventory (Adler 2025), which provides a systematically organised list of taxa with country-level or regional distribution records. It serves as the principal foundation for compiling regional species lists and corresponding distribution data. However, its worldwide scope limits its ability to resolve some identification uncertainties and fine-scale distribution patterns.

Checklists of blackflies are most often incorporated within broader Diptera inventories, as in those for Belgium (Lock 2018), the British Isles (Chandler 2024), Bulgaria (Hubenov 2021), Czechia and Slovakia (Jedlička and Kúdela 2025), Denmark (Petersen and Meier 2001), Finland (Ilmonen 2014), Hungary (Papp 2001), Italy (Ciadamidaro and Mancini 2022), Lithuania (Pakalniškis et al. 2006), Netherlands (Knoz and Beuk 2020), and Switzerland (López-Peña 2024). In some instances, the family is treated separately, as in Austria and Germany (Seitz 2022), Malta (Gatt 2015), Mordovia in the North Caucasus (Budaeva and Ruchin 2014), Poland (Niesiołowski 2007), Spain (López Peña and Jiménez-Peydró 2017), Ukraine (Sukhomlin and Zinchenko 2016), and the United Kingdom (López Peña and Cheke 2023). In other cases, blackflies are embedded in broader faunistic studies, as exemplified by Croatia (Ivković et al. 2016).

The aims of this study are:

- to critically examine and refine the existing data on European blackflies;
- to update and supplement summarised records of European simuliid species;
- to reassess the reliability of reported occurrences in Europe;
- to compile a list of species with unequivocally confirmed presence in Europe;
- to compile a list of species whose occurrence in Europe remains ambiguous.

## Data and methods

### Territory

The extent of the European territory considered in this study follows the delimitation adopted in Fauna Europaea (De Jong et al. 2015) and used in our study of blackfly chorotypes (Jedlička et al. 2024). This includes the European mainland and its islands, including the Macaronesian archipelagos (except the Cape Verde Islands), with the addition of the northern Caucasus (Soόs and Papp 1984–1994; Rubtsov and Yankovsky 1988; Kustov 2015).

The eastern boundary of Europe is conventionally defined along the Yugorsky Strait – Pay Khoy Ridge – Ural Mountains, and further south along the northern base of the Ustyurt Plateau and the North Aktau Ridge to the Mangyshlak Gulf (Chibilev and Bogdanov 2011). In cases of uncertainty or dispute, records from the Kara, Pechora, and Volga River basins are treated as European, whereas those from the Ob basin are considered Asian. For the continental boundary between Europe and Asia from the Caspian Sea to the Black Sea, we follow the main ridge of the Greater Caucasus (Rubtsov and Yankovsky 1988; Soόs and Papp 1984–1994; Abell et al. 2008; Kreft and Jetz 2010; Naseka 2010; Chibilev and Bogdanov 2011). From the Black Sea, the boundary extends through the Turkish Straits. In accordance with Fauna Europaea, all Mediterranean islands, Macaronesia, and the North Atlantic and Arctic islands are considered part of Europe (De Jong et al. 2015), even though they differ in their geographical and geological history.

The territorial units (OGU) applied in this study follow those used in the Inventory (Adler 2025). Differences from the units employed in the Catalogue (Rubtsov and Yankovsky 1988) are outlined in Suppl. material 2. No black fly records are available for San Marino, Monaco, or Vatican City (Ciadamidaro and Mancini 2022; Adler 2025). For the purposes of this study, Turkish Thrace—the European part of Turkey—is treated as a distinct operational geographic unit (Şirin et al. 2015).

### Data

This study is based on a structured evaluation of species-level taxa of European black flies. The workflow integrates multiple data sources and applies a stepwise filtering process to assess nomenclatural validity and distributional reliability. Data processing was carried out in two stages: data collection, and data assessment and filtering.

The study focuses on species recorded in Europe. Accordingly, the data of interest are species-level taxa with reported occurrence within the European territory, which can be expressed in the form ‘taxon name – occurrence record (Europe)’. Relevant data were extracted from both secondary sources (lists, catalogues, databases) and primary sources (original faunistic and taxonomic studies), particularly in cases where the latter had not been considered or had been misinterpreted in secondary compilations.

In the initial phase of our analysis, we relied primarily on three comprehensive secondary sources, collectively referred to as key comprehensive sources (KCS). These served as the foundation for compiling species-level taxa reported from Europe. Each source was systematically reviewed to extract taxon names and associated occurrence records. Where ambiguities, outdated nomenclature, or conflicting classifications were encountered, supplementary verification was conducted using primary literature.

The core data on the occurrence of extant species-level taxa were extracted from the 2025 edition of World Blackflies (Diptera: Simuliidae): A Comprehensive Revision of the Taxonomic and Geographical Inventory (Adler 2025; hereinafter referred to as **INV**)—the principal and most authoritative global source on blackfly taxonomy and distribution. These data were compared with the treatment of European blackflies in Fauna Europaea (Crosskey 2017; hereinafter **FaEu**), currently available in mapped form via the PESI portal (De Jong et al. 2015; PESI 2024). Where clarification was required or ambiguities arose regarding the identity or distribution of species or subordinate taxa, the information was further cross-checked against the Catalogue of Palaearctic Diptera (Rubtsov and Yankovsky 1988; hereinafter **CAT**). Together, these three sources—INV, FaEu, and CAT—provide a consolidated overview of blackfly taxa distributions, typically at the national scale, or, in the case of larger countries, at subnational or regional levels (cf. Jedlička et al. 2024).

**Figure 1.**
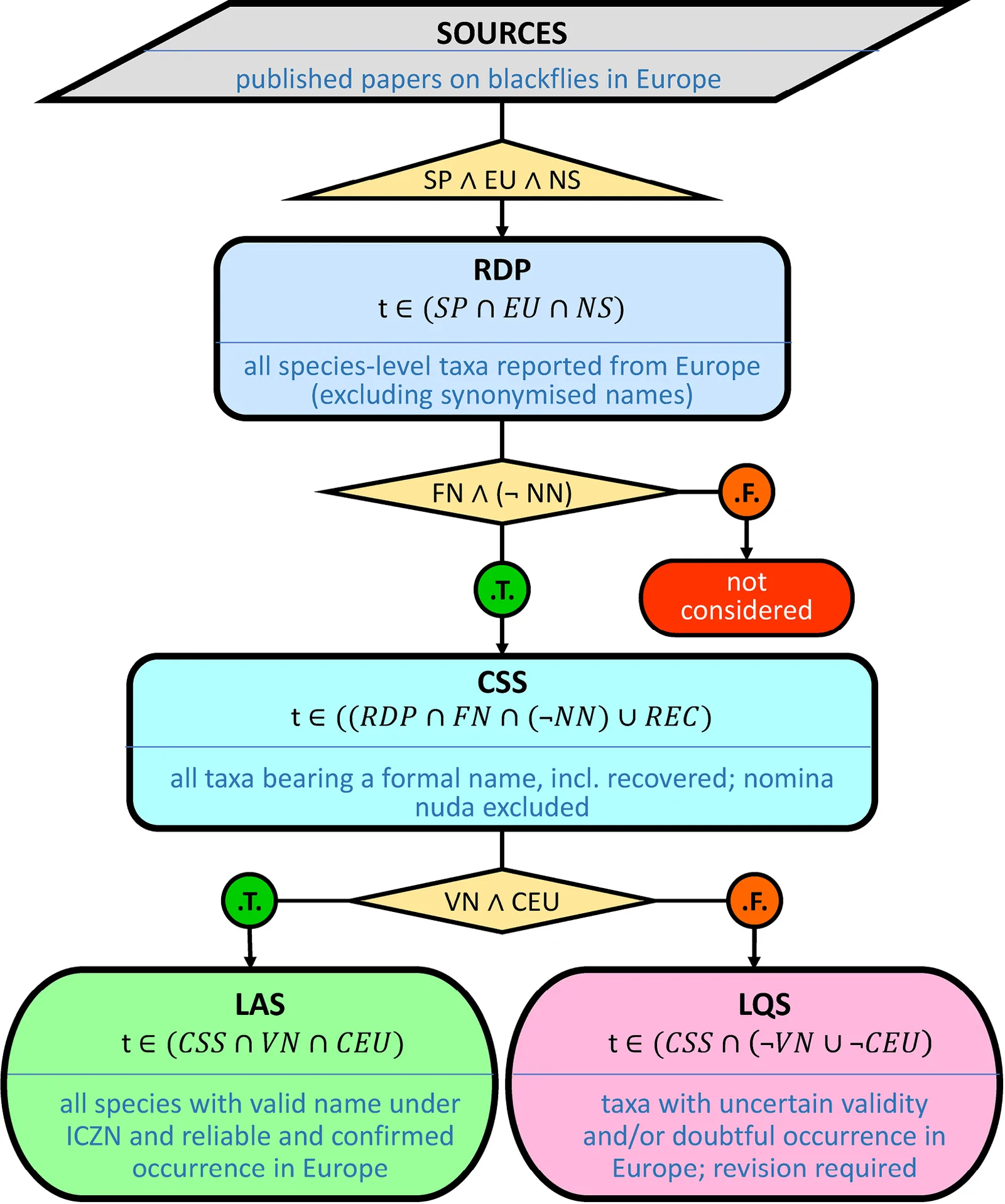
Workflow of data processing. Abbreviations: CEU – confirmed occurrence in Europe (recent and verified); CSS – core set of species: all taxa bearing a formal name (including those revalidated from synonymy; nomina nuda excluded); EUR – reported from Europe; FN – formally named; LAS – List of accepted species: taxa with a valid name under the ICZN and reliably confirmed occurrence in Europe; LQS – List of questionable species: taxa of uncertain validity and/or doubtful occurrence in Europe; NN – nomina nuda; NS – not synonymised; RDP – raw data pool: all species-level taxa reported from Europe (excluding synonymised names); REC – revalidated from synonymy; SP – species-level taxa; t – included taxa; VN – valid name (under ICZN).

In the next step, occurrence data for certain taxa—including some not listed in the KCS sources but reported from Europe—were supplemented with European records extracted from sources partly overlooked or not previously considered (cf. Jedlička et al. 2024). All species-level taxa recorded from Europe, whether listed in the KCS or other published literature, and not synonymised, were compiled into the raw data pool (RDP), which served as the basis for subsequent analytical processing (Fig. 1).

Printed sources covering European species and territories were digitised and converted into spreadsheet format (xlsx). The resulting dataset was disaggregated into individual species records, each associated with the operational geographic units (OGUs) representing the species’ distribution, and subsequently organised and analysed using standardised Microsoft Excel procedures (Microsoft Corporation 2021).

During the second work stage, all data were stepwise assessed, filtered, and sorted into subsequent sets.

In the first step, a refined dataset—the core set of species (CSS)—was derived by filtering the RDP. All taxa bearing a formal name were included, regardless of their current nomenclatural status, while *nomina nuda* were excluded. Additionally, species revalidated from synonymy were incorporated, and two taxa underwent individual re-evaluation.

In the second step, the CSS was divided into two subsets according to the following two criteria: i) nomenclatural validity (VN), i.e. correctness of the description and the validity of the name under the ICZN, including the existence or absence of a name-bearing type, the adequacy of the original description, a non-ambiguous taxonomic identity, and the need (or lack thereof) for revision, and ii) confirmed occurrence in Europe and reliability of identification (CEU), i.e. reliable confirmation of occurrence in primary sources, with particular attention paid to rare records not confirmed within the last 50 years, records from peripheral regions of the study area, and records in Europe that lie far outside the accepted distribution area of the species elsewhere.

Species meeting both criteria—being nomenclaturally valid under the ICZN and reliably confirmed in Europe by primary sources (VN ∧ CEU)—were considered reliably documented and included in the List of accepted European species (LAS). Species that did not meet at least one of these criteria were judged to require taxonomic, faunistic, identification, or zoogeographical revision and were consequently assigned to the List of questionable species (LQS).

## Results and discussion

One of the aims of this study was to compile an up-to-date list of simuliid species confirmed in Europe, based on an assessment of data reliability. Only extant taxa at the species level are considered; extinct, higher-level, and infraspecific taxa are not included in the scope of this study. Likewise, taxa and names currently treated as synonyms are not included.

All data are organised into four sets: the raw data pool (RDP); its descendant subset, enriched by revalidated taxa, the core set of European species (CSS); and two sets derived from the latter—namely, the List of accepted species (LAS) and the List of questionable species (LQS). These sets are defined and described sequentially and in greater detail below.

### Raw data pool

The raw data pool (RDP) comprises all species-level taxa reported from Europe representing the full spectrum of taxa potentially occurring in the region, irrespective of the certainty or validity of these records. Synonymised taxa are not considered.

The INV, the principal reference source, lists 259 such taxa, of which 112 have been identified as having additional data or corrections regarding their occurrence (see Suppl. materials 1, 2). A further nine species were incorporated into the RDP from other sources. FaEu includes 230 nominal species; after accounting for synonymy, 215 species remain. However, eight of these are listed as European simuliids solely on the basis of doubtful presence in one or two countries. The RDP therefore currently contains 268 extant blackfly species, although the varying quality of the data necessitated further refinement and cleaning. The number of taxa of the RDP may change due to the description of new species from Europe, the emergence of reliable records of a previously non-European species in Europe, synonymisation or revalidation of taxa, or the elevation of intraspecific forms to species level.

### Core set of European species

The core set of European species (CSS) was derived from the RDP by excluding nine taxa lacking a formal name and bearing only an informal designation like “aff. [species name], “sp. A”, “1”, “cf. *species*“, “morphotype 3“), etc.

Accordingly, two morphoforms were not included in the CSS: ‘1’ Kúdela & Jedlička, 2002 (*monticola* morphoform) from Slovakia, mentioned later as aff. *monticola* by Dušinský et al. (2006); and ‘II’ Grenier, 1953 (*Simulium* sp. ‘A’, attrib. Dorier, 1947). Neither has been described and formally named in accordance with the ICZN.

In addition, five cytoforms were excluded, ‘5’ Adler, 2016 (hypothetical species, *Prosimulium macropyga* species group), ‘Crete’ Procunier, 1982 (*Metacnephia*), ‘K’ Leonhardt, 1985 (*aureum* cytoform), ‘4’ (Chubareva & Petrova, 2003), ‘IL-8’ Adler, Belqat, González, Pérez & Seitz, 2016 (the latter two belonging to the *Simulium vernum* species group). These taxa were excluded because they lack a definitive status and a formal name, in accordance with the ICZN Code (Arts. 1.3.1 and 1.3.5).

**Figure 2.**
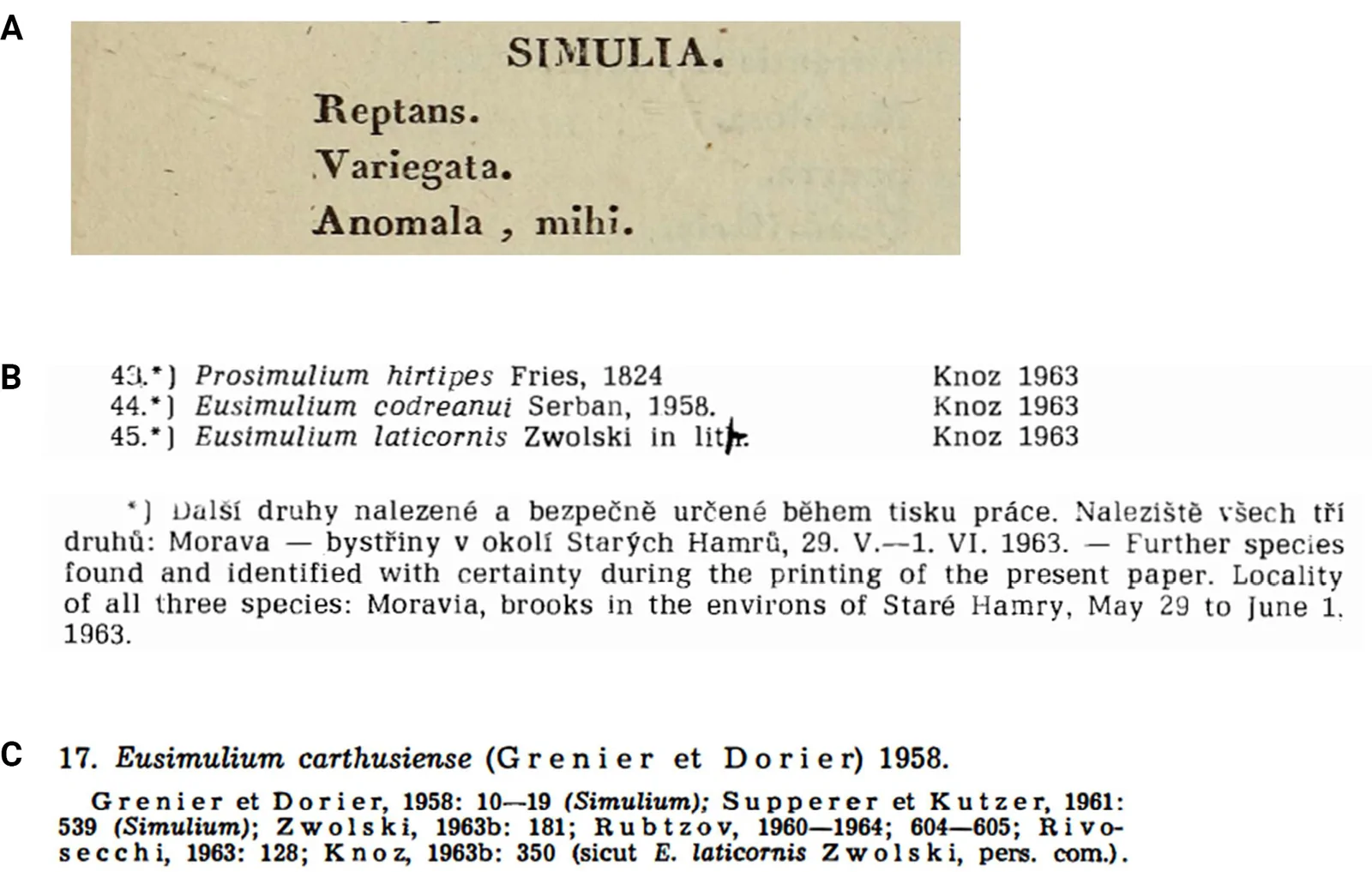
Nomina nuda in European blackflies. **A**. Original (non)-description of *Simulium anomalum* by Eversmann (1834); **B**. First mention of *Simulium laticornis* Knoz (attributed to Zwolski), without description (Knoz 1963); **C**. Informal synonymisation of *S. laticornis* Knoz (attributed to Zwolski) with *S. carthusiense* (Grenier & Dorier), 1958 in Knoz (1965), not repeated in Knoz (1980).

Furthermore, two nomina nuda reported from Europe were excluded: *S. anomalum* Eversmann, 1834, a typical nomen nudum lacking any description (Fig. 2), and *S. laticornis* Knoz, 1963 (attrib. Zwolski [correct author name]). The origin of the latter name is unclear. Knoz (1963) attributed it to Zwolski (as in “pers. com.”), whereas Zwolski (1963) stated that Knoz had informed him by letter about the “…presence of a new variant [of the species] in Czechoslovakia”. The name was never used by Zwolski in any published work. Knoz himself appears to have renounced its species status (Knoz 1980), referring to it only once (Knoz 1965) as a synonym of *S. carthusiense*, without providing a formal act of synonymisation (“Knoz 1963b: 350 (sicut *E. laticornis* Zwolski, pers. com.)”; see Fig. 2). Both names fail to meet the criteria set out in Articles 12 and 13 of the ICZN Code and are therefore unavailable. Consequently, they should be excluded from any list of European black fly species. This discussion is not intended for nomenclatural purposes; it serves only to illustrate the historical context.

As in our previous study on global chorotypes (Jedlička et al. 2024), we include in the CSS, as revalidated, two species: *Simulium annulipes* Becker, 1908 (cytoform C of *Simulium ruficorne* Macquart, 1838) and *Simulium beckeri* Roubaud, 1906 (cytoforms A1/A2 of *S. ruficorne* Macquart, 1838), known from the Mediterranean/Macaronesian region. Both species have recently been treated as subjective synonyms of *S. ruficorne* (e.g. Adler 2025). Based on chromosomal evidence, Cherairia and Adler (2018) provided well-documented support for the isolation of cytoforms A and C as separate species from the other, Afrotropical-origin cytoforms, a conclusion that is also consistent with zoogeographical considerations. They also indicated the possibility of their revalidation, noting that two of the five recently recognised cytoforms may correspond to species formerly placed in the synonymy of *S. ruficorne*. The most recent study by Adler et al. (in press) provided further evidence supporting this position, although the authors at the same time cautiously recommend postponing the establishment of *S. beckeri* as a distinct species until its isolation is definitively confirmed. We respect this view, but for pragmatic reasons we currently maintain the recognition of both named species as distinct for the purpose of this European species list. Should their synonymy be unequivocally demonstrated in the future, it will be considerably easier to merge distributional data than to separate them from an already generalised dataset.

Following a re-evaluation of the status of *Simulium aff. tuberosum*, previously treated in our study (Jedlička et al. 2024) as a species-level taxon, we merge here this form with *Simulium janzeni* Enderlein, in line with the views of Adler and Kuusela (1994) and Zwick (1995). Zwick found no morphological differences (in females) between *S. janzeni* and *S. tuberosum* (Lundström) and considered the former to represent a Central European species. Adler and Kuusela (1994), however, noted possible specific differences between *S. tuberosum* and material from southern Germany, suggesting that the latter may belong to *S. subtile* Rubtsov. This arrangement is provisional and does not affect the list of accepted species, as both *S. janzeni* and *S. subtile* are documented from Europe. The European members of the *tuberosum* species group need a revision and the number of species remains unresolved.

Following these adjustments, the CSS comprises 261 species. In the subsequent stages of the analysis, these were divided into two lists: the List of accepted species (LAS) and the List of questionable species (LQS).

### List of accepted species

The List of accepted species (LAS, see Suppl. material 1) was created from CSS as its subset comprising taxa fulfilling two principal criteria – validity under ICZN and a reliably confirmed occurrence in Europe. The occurrence in Europe must be supported by reliable primary records or consistent treatment across the KCS datasets. These species have clearly established taxonomic identities, comply with the provisions of the ICZN Code, and raise no substantive doubts regarding their validity. Most of them were originally described from European localities and are consistently recognised across relevant taxonomic sources.

Based on these criteria, we have included in the LAS 233 species-level taxa currently recognised from Europe. Sixteen of these are regarded as species complexes, suggesting that the actual number of species is likely to be higher.

The number of accepted species in Europe is influenced by how the boundary between Europe and Asia is drawn in the region between the Caspian and Black Seas. If the Manych Depression is taken as the boundary and the northern Caucasus is excluded from Europe, the number of species decreases by 14. Three of these are Caucasian endemics, while the remaining 11 reach their northern or north-western distributional limits in the Caucasus and are also known from other countries of southwestern and Central Asia; their ranges do not extend further into Europe.

The complete list of accepted species is presented in Suppl. material 1. In tabular form, each species entry includes its higher-level taxonomy, valid species name, distribution records as given in the INV, and, where applicable, any additional data or comments concerning distribution and occurrence.

### List of questionable species

The List of questionable species (LQS, see Suppl. material 2) represents a subset of the CSS and comprises taxa that fail to meet at least one of two principal criteria: validity under the ICZN and reliable, confirmed occurrence in Europe. These deficiencies distinguish them from the List of accepted species LAS. The LQS taxa can be further assigned into two groups.

The first group comprises taxa with unresolved taxonomic or nomenclatural status, or those failing to meet the requirements of the ICZN Code. This category includes species for which type material is absent ab initio, destroyed, or currently untraceable, as well as those whose original descriptions, illustrations, or indications do not permit reliable identification or differentiation from closely related taxa. In light of the absence of type material and the inadequacy of the original description to support unambiguous identification, such names may be considered nomina dubia in accordance with ICZN Articles 72.5.6 and 75.5.

In cases where type material exists but no holotype was designated, it may be necessary to select a lectotype in accordance with Article 74 of the ICZN Code. Where no original type material is available, the designation of a neotype may be considered under the conditions outlined in Article 75. Following appropriate redescription, the name may be confirmed or synonymised. If the preserved type material indicates that the name represents an older, valid synonym, and its reinstatement would disrupt nomenclatural stability, it would be advisable to initiate the process of declaring it a nomen oblitum, as permitted under Article 23.9 of the ICZN Code.

The second group comprises taxa whose occurrence in Europe is uncertain or insufficiently supported, primarily due to questionable identity, ambiguous identification, and associated zoogeographical uncertainties. Doubts about species identity and potential misidentification often stem from descriptions and/or illustrations that cast doubt on the reliability of the identification. Such discrepancies may arise when the descriptions and accompanying figures are inconsistent, diverge from the original source, or have been adopted from other authors—most notably Rubtsov (1956). This category also includes taxa listed in enumerative checklists without accompanying revisionary treatment or independent corroboration from other sources.

In many cases, doubts about identification are reinforced by distributional data. These often involve single or sporadic records from isolated peripheral regions well outside the species’ established range, which typically lies beyond the western Palaearctic. Such records provide insufficient evidence of a stable European presence and should be interpreted with caution. Taxa in this group should be flagged as questionable, pending re-examination of the original material or the availability of additional reliable records. Until such confirmation is obtained, they should not be considered part of the core European fauna.

The age of the record represents a distinct case, often intertwined with other issues, and pertains to historical records that have not been subsequently confirmed. Many such records consist of single-name entries from a lone site or appear in species lists lacking locality data and supporting documentation. These records may be over half a century old or more and remain unverified in primary studies, while secondary sources sometimes cite them as either reliable or doubtful. Notable examples are found in the Balkans (e.g. Bulgaria, Romania) and northern Russia. Historical records of this nature require priority verification through the re-examination of original material, critical evaluation of source data, and the search for new, reliable records before they are incorporated into the current European species list.

Records from individual countries are not evaluated if they fall within the polygon of the species’ European distribution (extent of occurrence) or concern endemics restricted to a defined area, as such cases do not influence the overall list of European species. However, if these are the only records available for a given taxon, they directly affect its inclusion and must therefore be assessed.

In general, we identified 28 species whose presence in Europe is currently considered questionable. Of these, 24 species require verification of their presence in the region, while the remaining four are taxonomically and nomenclaturally controversial and unresolved. The complete list of species is provided in the Suppl. material 2, which also contains the distributional data from INV, links to PESI, and a comparison with CAT. These species should be excluded from secondary syntheses unless their presence has been reliably confirmed by original studies or the relevant taxonomic and nomenclatural issues have been resolved.

Inclusion of a species/taxon in the LQS does not imply that its occurrence in Europe is impossible; rather, it reflects the current lack of unequivocal supporting evidence. A single reliable record may suffice to confirm a species’ presence in Europe, yet its exclusion cannot be definitively proven—only inferred. Consequently, removal from the LAS requires careful evaluation of multiple factors. The rationale for excluding each species and assigning it to LQS is provided in detail on a case-by-case basis.

The only primary sources reporting the occurrence of *Helodon rubicundus* Rubtsov, 1956 in Europe appear to be publications by the Perm School: Bel’tyukova (1962) from the Perm region, Bel’tyukova and Shubina (1977) from the southern Urals (both CET = CR), and Shubina (1986) from the Shchugor basin (NET = NR). However, other relevant sources report only *H. ferrugineus* (Wahlberg) from these areas (Rubtsov 1956, 1959–1964; Usova 1961; Patrusheva 1982; Sharkov et al. 1984; KCS), while the occurrence of *H. rubicundus* is restricted exclusively to east Siberia and the Russian Far East (Rubtsov 1959–1964); its presence in Europe therefore requires re-evaluation and confirmation based on verified material.

Most sources indicate the range of *Prosimulium tridentatum* Rubtsov, 1940 in east Siberia, Mongolia, and the Far East (Rubtsov 1959–1964; Patrusheva 1982; Petrozhitskaya and Rodkina 2009a, 2009b; KCS), as well as west Siberia (Altai Mts., and Khakassia). According to Aibulatov (2015), citing several earlier works, the species also occurs in northern Russia. The species has been reported from the Shchugor Basin in European northern Russia (Shubina 1986; Ostroushko et al. 2007), with both records ultimately tracing back to Ostroushko and Bel’tyukova (1983, non vidi). No new primary data are available, and subsequent publications repeat the original records.

*Prosimulium ventosum* Rubtsov, 1956 was originally described as a form of *P. macropyga* based on larvae from the Baikal region (Rubtsov 1956, 1959–1964). It was later reported as *P. macropyga ventosum* by Bel’tyukova (1962) from tributaries of the Chusovaya River, and as a Uralo-Siberian boreal species from Komi (northern Russia), although these records rely solely on larval morphology (Ostroushko and Bel’tyukova 1983). Notably, Bel’tyukova and Shubina (1977) and Shubina (1986) did not mention the species. More recently, Yankovsky and Yankovsky (2006) considered the species to be a synonym of *macropyga*.

*Metacnephia persica* (Rubtsov, 1940) is one of three *Metacnephia* species reported in Europe solely from Romania (Dinulescu 1966), and for which there are serious doubts regarding the correctness of the identification (cf. Stloukalová and Jedlička 2007). The distribution of *M. persica* includes Lebanon, Armenia, Azerbaijan, Iran, Turkmenistan, Uzbekistan, and Kazakhstan (INV). Dinulescu’s descriptions of adults are based on Rubtsov (1956), which suggests that he recorded only preimaginal stages in Romania. The figure of larval characters is likewise derived from Rubtsov (1956). Dinulescu’s description of pupae includes two characters—a solid cocoon with a slightly raised anterior margin and a gill with 20–22 respiratory filaments—presumably forming the basis for his identification. However, both these characters are variable and similar in other species of the genus. Considering that Dinulescu (1966) did not record *M. danubica*, *M. blanci*, and *M. uzunovi*, the evidence for the occurrence of *M. persica* in Romania is insufficient.

*Metacnephia ramificata* (Rubtsov, 1956) was described from the Siberian Far East, and later recorded in Mongolia and Uzbekistan. The only European record is from Romania (Dinulescu 1966). He provides the descriptions of all stages, but those of males and larvae are entirely based on Rubtsov (1956), indicating that he did not record these stages himself. The description of the female, which focuses mainly on the antennae and mouthparts, cannot serve as a basis for identification, since Rubtsov (1956) did not describe the female and considered it unknown. Therefore, the only stage by which the species could be identified in Romania is the pupa. However, the characters given are either variable and insufficient for species identification (shape and number of the gills) or directly contradict the original description (cocoon shape). We consider the evidence for the occurrence of *M. ramificata* in Romania to be insufficient.

*Metacnephia sommermanae* (Stone, 1952) has a Holarctic distribution, ranging from Yukon and Alaska through Siberia to Kazakhstan and Uzbekistan (INV). The only European record is from Romania (Dinulescu 1966), under the name *crassifistula* (Rubtsov). His descriptions of males and larvae are entirely based on Rubtsov (1956), indicating that he sampled only pupae. However, the pupal characters described by Dinulescu (1966) do not provide sufficient evidence for the occurrence of *M. sommermanae* in Romania.

Simulium (Eusimulium) brachyantherum Rubtsov, 1947 was originally described from a mountain spring in Tajikistan and subsequently reported from Armenia, Iran, Romania, and Uzbekistan (INV). The Armenian record (Rubtsov 1956) was later regarded as erroneous (Terteryan 1968) and Rubtsov (1959–1964) restricted the species’ distribution to Central Asia. Dinulescu (1966) reported the species from Romania and Bulgaria, but its presence in Bulgaria (Vidin region) could not be confirmed (Kovachev 1979). Crosskey (2004, 2017) considered the Balkan records doubtful, and only the Romanian record is retained in the INV. Confusion or misidentification with another species from the subgenus *Eusimulium* cannot be ruled out.

The occurrence of Simulium (Nevermannia) ruficorne Macquart, 1838 (complex)—originally described from Réunion and with a confirmed Afrotropical distribution—is considered doubtful in Europe. Cytological evidence revealed three distinct cytoforms in Europe, forming separate clades. Two of them (A1/A2 and C) correspond to S. (N.) beckeri Roubaud, 1906 and S. (N.) annulipes Becker, 1908, respectively (Cherairia and Adler 2018).

Although no formal taxonomic reassignments were proposed and the matter has not yet been definitively resolved (Adler et al. 2026), both taxa are treated here as valid and accepted members of the European fauna. In contrast, European records of *S. ruficorne* from Malta, Portugal, and Spain lack morphological, cytological, or molecular support. Pending further evidence, *S. ruficorne* is provisionally excluded from the list of confirmed European species.

The only European record of Simulium (Nevermannia) fluviatile Radzivilovskaya, 1948 is from Romania (Dinulescu 1966); however, the descriptions and illustrations provided differ markedly from Rubtsov’s (1956) description in several key characters. Moreover, the Romanian locality is strongly disjunct from the species’ known range in the eastern Palaearctic. This record is included in INV, but absent from FaEu/PESI, and considered doubtful in the CAT. Rubtsov (1959–1964) gave the distribution as Asia orientalis.

Simulium (Simulium) alajense Rubtsov, 1938 is a species distributed across Palaearctic Asia, extending westward to Iran, Armenia, and Turkey (CAT); in Europe, it is listed from Bulgaria and as a questionable species from Romania (INV). However, we did not find any primary records for the territory of Romania. The existing record of *Simulium alajense hiemale* (Rubtsov, 1938) from Romania by Dinulescu (1966, in genus *Tetisimulium*) should be treated as *Simulium hiemale* (Rubtsov, 1956). Therefore, the only European record of the species is from Bulgaria (Hubenov 2021, citing Kovachev 1969 – non vidi). The species is not listed from Europe in CAT or by Yankovskiy (1993).

Simulium (Simulium) desertorum Rubtsov, 1938, described from northern Tibet, is known from Central Asia, with its westernmost occurrence in Kazakhstan. The Bulgarian record (Hubenov 2021, citing Kovachev 1969 – non vidi) is not taken into account in CAT, is listed as doubtful in FaEu, and is considered present in INV. Yankovskiy (1993) in his revision of the type material of *Tetisimulium* species indicates the distribution of *S. desertorum* in Central Asia, Kazakhstan, and Altay. The probability of its occurrence in Bulgaria is low.

Simulium (Simulium) hiemale (Rubtsov, 1956) was recorded in Transcaucasia and Uzbekistan (INV). It was reported from Romania as *Simulium alajense hiemale* (Rubtsov, 1938) by Dinulescu (1966), who probably found only larvae, as his descriptions of adults and pupae are based on Rubtsov (1956). The species is listed from Romania as well as Bulgaria in CAT, but is completely missing from Europe in INV.

Simulium (Simulium) kerisorum (Rubtsov, 1956) was reported in Europe only from Bulgaria (Hubenov 2021, citing Kovachev 1969 – non vidi). This record is not included in CAT, considered doubtful in FaEu, but accepted in INV. The species was described from Azerbaijan and has also been repeatedly reported from Turkey (e.g. Şirin et al. 2014; Başören and Kazanci 2016). In light of these records, its occurrence in Bulgaria as part of a Centralasian-Turano-Euxinian distribution (Jedlička et al. 2024) cannot be excluded. However, given the unresolved suspicious synonymy with *S. bezzii* (Corti) (cf. Crosskey and Zwick 2007), we regard *S. kerisorum* as a questionable species for Europe.

Simulium (Simulium) latimentum (Rubtsov, 1956) was described based on larvae from Kyrgyzstan and reported from western Siberia and Mongolia (Rubtsov 1959–1964; Yankovskiy 1993, INV). All KCS treat the Romanian larval record as doubtful, and omit the single Bulgarian record (Beron 2004; Hubenov 2021 citing Kovachev 1975 – non vidi).

Simulium (Simulium) bimaculatum (Rubtsov, 1956) is accepted in INV for Uzbekistan and Kazakhstan, whereas records from China and Romania are considered doubtful. Rubtsov (1959–1964) reported the species distribution in the Urals, Siberia, and of some “forms” in Central Asia and China. Dinulescu (1966), who reported the species from Romania, provided descriptions and illustrations of males, females, and pupae (not originally described), yet many of these features differ from Rubtsov’s (1956) original description. The Romanian record—the only known European occurrence—is regarded as doubtful across all KCS.

Simulium (Simulium) malyschevi Dorogostaisky, Rubtsov & Vlasenko, 1935, otherwise known from the eastern Palaearctic and western Nearctic, is not listed from Europe in any KCS. The identity of the only reported European records—from northern European Russia (NET) published by Bel’tyukova and Shubina (1977), Sharkov et al. (1984), Shubina (1986), and Ostroushko et al. (2007), and later referenced by Aibulatov (2016)—remains unresolved and requires further verification.

Simulium (Simulium) deserticola Rubtsov, 1940 was originally described from the semi-desert basin of the Khovd River (NW Mongolia), with a neotype later designated from Tajikistan. According to INV, its distribution includes Tajikistan, Mongolia, Romania, Ukraine, and Uzbekistan. Rubtsov (1959–1964) reported the species range in Central Asia. However, the Romanian record is considered doubtful in FaEu, and the occurrence in Ukraine is not listed there. A Bulgarian record (Hubenov 2021 citing Russev et al. 1994) is likewise absent from both FaEu and INV.

The history of Ukrainian records is complex. The first mention by Lebedeva (1970 – non vidi) was repeated by Usova (1974, 1987). Material from Ukraine is present in the collection of the Department of Zoology, Lesya Ukrainka Eastern European National University, Lutsk (five specimens: 2 larvae, 1 pupa, 1 male, 1 female; Sukhomlin and Zinchenko 2018); the species has not been sampled in recent years (K. B. Sukhomlin, pers. comm. 28 Feb. 2018). More recently, the species was reported from two contrasting habitats: large rivers in Ukrainian Polissya (Kaplich et al. 2015), and spring streams of the Severskiy Donets basin (Reva et al. 2012, 2013).

While these authors noted general agreement with Rubtsov’s description, they also pointed out morphological differences in both adults and larvae. Discrepancies with available identification keys further complicate the interpretation of these records.

Given that a species originally described from a semi-desert river basin in Mongolia and subsequently reported from Central Asia is unlikely to occur in both spring streams and large rivers of European mixed-forest regions (cf. Reva et al. 2013; Kaplich et al. 2015) and considering the taxonomic uncertainties within the ornatum group, we concur with K. B. Sukhomlin (pers. comm. 28. 2. 2018) that the European material assigned to *S. deserticola* requires revision. Under these circumstances, we have excluded the species from the list of accepted European taxa, despite its traditional inclusion in more recent publications (Panchenko 2016; Sukhomlin and Zinchenko 2016).

The identity of Simulium (Simulium) simoffi (Enderlein, 1924) is doubtful. The type specimen–the only known female–is lost (destroyed), with only the pin and labels preserved (Zwick 1995; Werner 1996). Rubtsov treated it inconsistently: as a synonym of *S. baracorne* Smart (Rubtsov 1959–1964, supported by one of the labels on type pin – see Zwick 1995), but also as a valid species (Rubtsov 1956, 1959–1964). Based on the original description of *simoffi* by Enderlein and *ruficorne* by Baranov, Zwick (1995) concluded that *simoffi* resembles *S. trifasciatum* Curtis, whereas Werner (1996) indicated synonymy with *S. ornatum* Meigen. Rubtsov’s interpretation, synonymising it with *S. baracornis*, would violate the nomenclatural stability; Zwick (1995) therefore considered it a nomen dubium. Despite this, the species is listed as valid in KCS. The appropriate solution would be to designate a neotype from newly collected material at the type locality. Beyond Enderlein’s original description, the species has been mentioned only by three other Bulgarian authors in the 1920s (see Hubenov 2021) and by Russev et al. (1994). No further primary sources have been found. We therefore follow Zwick’s opinion.

Simulium (Simulium) pavlovskii Rubtsov, 1940 is known from Siberia (INV: FE incl. Sakhalin, ES, in WS from Altai), as well as from China and Mongolia. It is not listed as present in Europe in CAT, FaEu, or Rubtsov 1959–1964). A record from Ukraine, marked as doubtful in INV, probably derived from Usova (Usova 1987, earlier original sources not seen), who mentioned the species as sporadic in the [Ukrainian] Carpathian Mts. More recent revisions (Panchenko 2016; Sukhomlin and Zinchenko 2016) consistently state that the species is absent from Ukraine. We therefore omit it from the list of European species.

Simulium (Simulium) kurense Rubtsov & Djafarov, 1951 is not included in any KCS for the territory of Europe; Rubtsov (1959–1964) reported the species from the Caucasus. In Europe, this species was reported—only once from Bulgaria—in a hydrobiological study by Kovachev (1976), who mentioned larvae and pupae without accompanying descriptions; the illustrated features do not fully correspond to Rubtsov’s (1956) description. All subsequent sources merely repeated this record (Russev et al. 1976; Islam et al. 1986; Hubenov 2021) or even omitted the species from the initial location (Uzunov et al. 2011).

Simulium (Simulium) parvum Enderlein, 1921 was originally described based on a single female holotype collected in Europe (details unknown, Zwick 1995; Werner 1996). A single second specimen, later identified by Enderlein from Lapland, was identified by Rubtsov (labelled in 1958, see Zwick 1995) as possibly belonging to *S. galeratum* Edwards; no further records are known. Subsequently, Rubtsov (1959–1964) provisionally assigned a male to this species, but suggested that it may be a new species close to *S. truncatum* (Lundström). According to Zwick (1995), the species is a member of the *reptans* group and differs from other members by the structure of the spermatheca. We regard this taxon as *species* inquirenda and have included it in the List of questionable species, pending further taxonomic clarification taking into account the scheme presented above in an earlier paragraph in this section.

Simulium (Simulium) exile (Rubtsov, 1956) was originally described from Azerbaijan, which remains its confirmed distribution area in both FaEu and CAT, Rubtsov (1959–1964) declared its range as Transcaucasia; being also recorded from Kazakhstan (INV). The only European record accepted by all KCS is from Romania. However, Dinulescu’s (1966) descriptions of males, females, and pupae do not fully correspond to Rubtsov’s (1956) original description. The reports from Romania require verification (Kúdela 2006), as does the record from Northern Russia (Sharkov et al. 1984), since confusion with members of the *variegatum* species group is conceivable.

Simulium (Simulium) bergi Rubtsov, 1956, described from Georgia, was long regarded as a Caucasian endemic (Rubtsov 1959–1964; Terteryan 1968) until it was reported from Anatolia (Crosskey and Zwick 2007; Adler et al. 2016b). Surprisingly, it has not yet been recorded from the North Caucasus (Budaeva and Khitsova 2016). The species has been repeatedly reported from continental Ukraine, particularly in lowland regions (Panchenko 2003, 2012, 2016; Kaplich et al. 2011, 2014, 2015; Sukhomlin and Zinchenko 2018), a distribution accepted in the INV; the older FaEu and CAT understandably could not reflect these findings. However, Ukrainian records differ in several respects from Terteryan’s original description of adults and larvae (in Kaplich et al. 2015: figs 108a, 108b; figured only, with no description), as well as in the larval breeding sites: in Armenia, the species was found in fast-flowing high-elevation rivers within gorges, whereas in Polesia it was collected from small- and medium-sized lowland rivers. Confusion with another species of the group cannot be ruled out, and the species identity requires verification.

Simulium (Simulium) curvitarse Rubtsov, 1940 was originally described from the Russian Far East, with its accepted distribution including the Far East and China. A record from the Leningrad region (Aibulatov 2013), based on collection material, lies far outside this range and requires verification.

Simulium (Wilhelmia) turgaicum Rubtsov, 1940 was originally described from Kazakhstan and has been reported from large parts of Asia, ranging from Turkey in the west to China in the east. However, four European countries are also listed in the published sources—Ukraine (Usova 1987), Slovenia, Bosnia and Herzegovina (Đuknić et al. 2019), and Czechia (Šikutová et al. 2026)—and these records are likewise reflected in the INV. Sukhomlin and Zinchenko (2016) listed *S. turgaicum* among the species whose occurrence in Ukraine they were unable to confirm. They reviewed the slides on which Usova (1987) based her report of this species from the steppe zone of Ukraine and identified them as *S. lineatum* (Meigen). The recent records of *S. turgaicum* from Slovenia, Bosnia and Herzegovina (Đuknić et al. 2019), and Czechia (Šikutová et al. 2026) are also doubtful. The presence of *S. turgaicum*—one larva from the Una River in Bosnia and Herzegovina, one pupa from the Sava River in Slovenia, and females from Lednice in south Moravia (Czechia)—is evidently based solely on mitochondrial COI sequences. In this case, we cannot accept the occurrence of a species based only on the sequence of part of a single mitochondrial gene as reliable evidence. The three closely related species, *Simulium balcanicum* (Enderlein), *S. turgaicum*, and *S. lineatum* show very similar COI sequences, and the phylogenetic tree based on COI presented by Đuknić et al. (2019) does not support monophyletic groups corresponding to the species; instead, it reveals a mixture of lineages, most of which lack sufficient statistical support. Moreover, the authors completely ignore other possible explanations for shared mitochondrial DNA sequences among different lineages (species), such as incomplete lineage sorting or hybridisation—both rather likely within a complex of closely related species with only minor morphological and chromosomal differences. The presence of the species in Europe was not confirmed even in the cytotaxonomic study by Adler et al. (2015), who delimited the range of the species to southwestern Asia, from Turkey to Central Asia.

*Simulium canescens* Bremi in Kölliker, 1842, is considered valid but unplaced within *Simulium* (INV); CAT treats it as nomen dubium, and FaEu does not mention it. López-Peña (2024) corrected the spelling of the author’s name according to the original source and documented the existence of type material, a finding later confirmed by Adler and López-Peña (2025). Some uncertainty nevertheless remains regarding the authorship of the species name *canescens*. Kölliker (1842: 11, footnote) explicitly stated that the species was named by Bremi: “Species novam, cui … Bremi nomen imposuit” [“a new species, to which … Bremi has given the name”]. According to Article 12.1 of the International Code of Zoological Nomenclature (ICZN), however, “every new name published before 1931 … must be accompanied by a description or a definition.” In the same sentence, Kölliker continues, “… brevi hic describam” [“… I will briefly describe it here”], which suggests that Bremi proposed the name, whereas Kölliker provided the description. If this interpretation is adopted, the correct authorship should be attributed to Bremi & Kölliker under Article 50.1 of the ICZN. Consequently, the appropriate name becomes *Simulium canescens* Bremi & Kölliker, 1842 in Kölliker, 1842.

A separate issue concerns the identity of the species. Based on the original description, Adler and López-Peña (2025) reasonably concluded that the name may refer to either *S. equinum* or *S. lineatum*, and they synonymised it with the latter. This conclusion is acceptable, though not entirely convincing. The possibility outlined by those authors—re-examining the type material, designating a lectotype, and undertaking additional nomenclatural actions—may, in light of current knowledge, prove necessary.

*Simulium incanum* Loew, 1840 was described (Loew 1840) in Oken’s Isis, once an influential interdisciplinary periodical, whose publishing history was repeatedly affected by censorship and political suppression, which contributed to its limited accessibility (Bachleitner 2022). CAT lists it as a valid species with the distribution “Europe: PL [= Poland]”, while INV treats it as unplaced within *Simulium*, though with the same distribution. In FaEu/PESI the species is omitted. The description—barely fifty words (eight for the actual diagnosis)—is entirely devoted to the colouration of a single female, assumed to be typical and species-identifying. The specimen has not been found in the Loew’s collection at the Museum zur Naturkunde, Berlin (Werner 1996), nor is it listed in the databases of the Natural History Museum in Dublin (A. O’Hanlon, pers. comm. 6 June 2025), the Natural History Museum London (Crosskey and Lowry 1989; Natural History Museum 2014), the Swedish Museum of Natural History, Stockholm (Naturhistoriska riksmuseet 2025), or the Oxford University Museum of Natural History (R. Douglas, pers. comm. 6 June 2025). Based on the available evidence, we assume that Loew’s original specimen is lost. Although the author considered the description sufficient, it is inadequate for unambiguous species identification. Accordingly, we regard the name as a nomen dubium. Further action may follow a course similar to the scheme presented above in an earlier paragraph of this section.

*Simulium lividum* (Schellenberg & anonymi, 1803), originally described as *Hirtaea livida* Schellenberg & anonymi, 1803 from Switzerland, has subsequently been listed among *Simulium* species unplaced to subgenus (Crosskey 1988; Crosskey and Howard 1997; INV). In CAT, the species name *livida* appears only in a single remark—“livida: Meigen, 1818: … not *Hirtea* [original: *Hirtaea*, recent study] *livida* Schellenberg, 1803“—where it is treated as a synonym of *Odagmia variegata* (Meigen). Schellenberg’s *livida* is otherwise absent from CAT as well as from FaEu/PESI.

Schellenberg’s figures are accompanied by a text—written by two anonymous “Liebhaber der Entomologie” [= entomology enthusiasts], as stated by Schellenberg—that briefly describes several characters of the species. The authorship could therefore be attributed to Schellenberg & anonymi. Because both the figures and the accompanying text are insufficient for confident identification, and the original specimens are considered lost (López-Peña 2024; Adler and López-Peña 2025), López-Peña (2024) proposed treating *S. lividum* as a nomen dubium, a view we follow. Adler and López-Peña (2025) later suggested synonymising *S. lividum* with *S. reptans* as the least disruptive solution to maintain nomenclatural stability, a proposal that appears reasonable. Should the original specimens—however unlikely—be recovered, the scheme outlined earlier in this section would apply.

### Higher taxa

For higher taxa, the taxonomic and family system employed herein follows that of INV, with taxa within each higher-level taxon arranged alphabetically. At the level of species groups—defined as aggregates of species below the subgeneric rank—we adhere strictly to Article 6.2 of the International Code of Zoological Nomenclature, which stipulates that such names “must always begin with a lower-case letter and be written in full” (ICZN 1999).

All known European blackfly species belong to the subfamily Simuliinae (Table 1); the other subfamily, Parasimuliinae, is exclusively Nearctic. The Simuliinae in Europe are represented by both recognised tribes: the Holarctic Prosimuliini (4 genera, 21 species) and the cosmopolitan Simuliini (5 genera, 212 species). In total, nine genera (of 31 worldwide), 14 subgenera (of 51) and 22 species-groups (of 126) are represented in Europe; one additional species group, *bimaculatum*, represented by *S. bimaculatum* (Rubtsov), is currently recorded in LQS only.

**Table 1.** Number of accepted and questionable species in higher taxa.

**Systematic categories**:					**species number**		
**subfamily**	**tribe**	**genus**	**subgenus**	**species group**	**accepted**	**questionable**	**total**
Simuliinae Newman					233	28	261
	Prosimuliini Enderlein				21	3	24
		*Helodon* Enderlein			1	1	2
			*Helodon* Enderlein		1	1	2
		*Prosimulium* Roubaud			16	2	18
				*hirtipes* (Fries)	13	1	14
				macropyga (Lundström)	3	1	4
		*Twinnia* Stone & Jamnback			1	-	11
		*Urosimulium* Contini			3	-	3
	Simuliini Newman				212	25	237
		*Cnephia* Enderlein			3	-	3
		*Greniera* Doby & David			4	-	4
				*fabri* Doby & David	4	-	4
		*Metacnephia* Crosskey			11	3	14
		*Simulium* Latreille			192	22	214
			*Boophthora* Enderlein		1	-	1
			*Boreosimulium* Rubtsov & Yankovsky		7	-	7
				*annulus* (Lundström)	3	-	3
				*baffinense* Twinn	4	-	4
			*Byssodon* Enderlein		1	-	1
				*meridionale* Riley	1	-	1
			*Eusimulium* Roubaud		13	1	14
			*Hellichiella* Rivosecchi & Cardinali		8	-	8
				*congareenarum* (Dyar & Shannon)	8	-	8
			*Montisimulium* Rubtsov		2	-	2
			*Nevermannia* Enderlein		54	2	56
				*ruficorne* Macquart	10	1	11
				*vernum* Macquart	45	1	46
			*Obuchovia* Rubtsov		9	-	9
			*Psilozia* Enderlein		1	-	1
			*Rubzovia* Petrova		3	-	3
			*Schoenbaueria* Enderlein		10	-	10
			*Simulium* Latreille		72	15	87
				*argenteostriatum* Strobl	2	-	2
				*bezzii* Corti	1	5	6
				*bukovskii* Rubtsov	3	-	33
				*bimaculatum* (Rubtsov)	-	1	1
				*malyschevi* Dorogostaisky, Rubtsov & Vlasenko	4	1	5
				*noelleri* Friederichs	4	-	4
				*ornatum* Meigen	13	2	15
				*pavlovskii* Rubtsov	1	1	2
				*reptans* (Linnaeus)	8	2	10
				*slossonae* Dyar & Shannon	1	-	1
				*tuberosum* (Lundström)	10	-	10
				*variegatum* Meigen	10	1	11
				*venustum* Say	15	2	17
			*Wilhelmia* Enderlein		10	1	11
				*equinum* (Linnaeus)	10	1	11
			unplaced to subgenus		-	3	3
		*Stegopterna* Enderlein			2	-	2

### Faunal composition and connections

A total of 2,415 extant named species are currently recognised worldwide (INV). In this study, we accept 233 species with confirmed occurrence in Europe, representing less than 10% of the global fauna.

**Figure 3.**
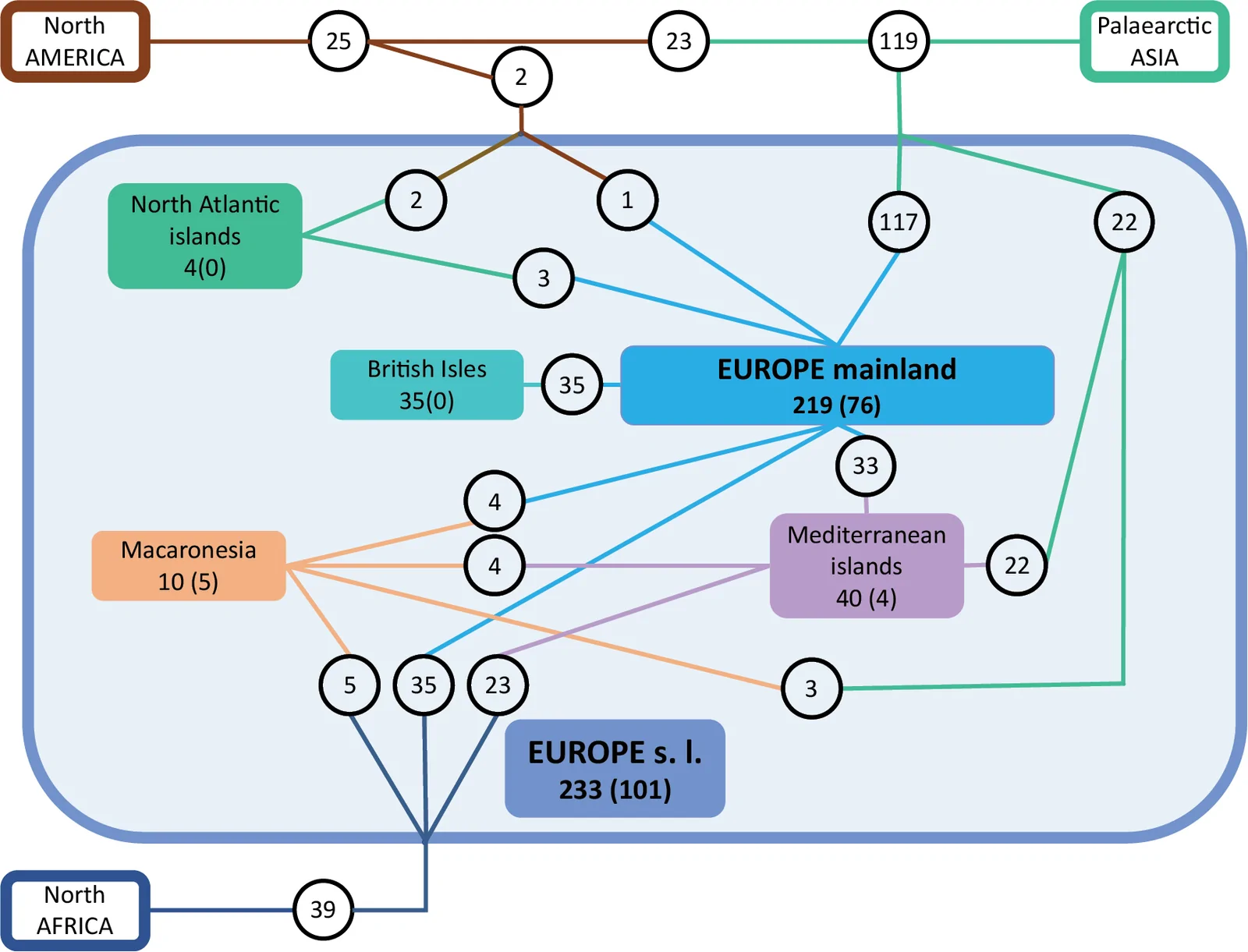
The number of species in different parts of Europe (shown in quadrilaterals) and the number of shared species between these areas and adjacent regions (shown in circles).

Of the accepted species, Europe *sensu lato* (mainland and islands) shares 133 species (57.1%) with other continents, while 100 species (42.9%) are endemic (Fig. 3). These endemic species exhibit considerable variation in the extent of their distribution, ranging from large portions of the continent (e.g. *S. argyreatum* Meigen) to a single island or mountain range (e.g. *S. timondavidi* Giudicelli from Corsica, *M. sardoa* (Rivosecchi & Contini) from Sardinia, *S. joanae* Seitz, Zwick & Adler from Madeira, *S. hasekei* Seitz, Adler & Remschak from the Alps).

Europe shares 119 species (51.1%) with the Palaearctic Asia, including the Sibero–European species inadvertently omitted in the previous study (Jedlička et al. 2024). Of these, 117 species (50.2% of the pan-European fauna and 53.4% of mainland species) occur on the mainland, and 36 are also recorded from the Mediterranean islands. Only one of these species, *S. ibleum*, known from Asia (Lebanon, Turkey), is recorded from the Mediterranean islands but absent from the mainland. Four species of this group—*S. petricolum* (Rivosecchi), *S. beckeri*, *S. intermedium* Roubaud, and *S. pseudequinum* Séguy—have been recorded from Macaronesia, all of which also occur in continental Europe. Iceland harbours only two European–Asian species (*S. aureum* Fries, *S. vernum* Macquart) and is the only North Atlantic island where both occur.

Twenty-five species (10.7%) are shared with North America, representing a subset of the Holarctic chorotype. Only two—*S. vittatum* Zetterstedt and *S. usovae* (Golini)—are absent from Asia, forming the American–European chorotype. *Simulium vittatum*, known from mainland North America, Greenland, Iceland, and the Faroe Islands, is absent from the European mainland and all other European islands, whereas *S. usovae* is restricted in Europe to Scandinavia. The remaining 23 species also occur in Asia.

An additional 39 species (16.7%) are shared with Palaearctic Africa, 35 of which (16.0%) occur on the mainland. Three species–west Mediterranean–Macaronesian S. (E.) velutinum (Santos Abreu), Pan-Mediterranean *S. ibleum* (Rivosecchi), and west Mediterranean *S. ichnusae* Rivosecchi & Contini–are absent from the mainland. European islands share 30 species with Africa, 23 of them on Mediterranean islands and five in Macaronesia.

In mainland Europe, 219 species have been recorded, of which 163 (74.4%) do not occur on European islands. The mainland shares 117 species (53.2%) with Asia, 36 species (16.9%) with Africa, and 25 species (11.4%) with North America. A total of 76 species are endemic to the European mainland, representing 32.6% of the total European fauna and 34.7% of the continental fauna.

A total of 70 species are recorded from European islands, 56 of which (80.0% of insular species) are shared with the mainland, 37 (52.9%) with Asia, 30 (42.9%) with Africa, and four (5.7%) with America. The highest number of species occurs on the Mediterranean islands (40 species, 33 of which also occur on the mainland; 36 in the western Mediterranean and 14 in the eastern Mediterranean), followed by the British Isles (35 species, all shared with the mainland), Macaronesia (10 species, four shared with the mainland), and the North Atlantic islands (four species, three shared with the mainland). Nine species are restricted to European islands. Four of these occur exclusively on Mediterranean islands (*S. timondavidi* on Corsica; *S. sardoa* and *S. continii* (Rivosecchi and Cardinali) on Sardinia; and *S. flexibranchium* Crosskey on several Greek islands), and another five in Macaronesia (*S. azorense* (Carlsson) on the Azores, *S. joanae* on Madeira, *S. guimari* Becker, *S. annulipes* and *S. paraloutetense* Crosskey on the Canary Islands). Four further species are insular in Europe, but have broader distributions: *S. velutinum* and *S. ichnusae* also occur in Africa, *S. ibleum* occurs in both North Africa and western Asia (Lebanon and Turkey), and *S. vittatum* is restricted in Europe to Iceland and the Faeroe Islands, though its range is centred in North America.

At the species level, the European fauna is predominantly Palaearctic, with 208 species (89.3%) whose ranges lie within this realm, while only 25 species (10.7%) belong to Holarctic chorotypes. Among the Palaearctic species, 37 (15.9%) have wide distributions—Pan-Palaearctic, Euro-Asian, or Sibero-European chorotypes—whereas the remaining 170 species (73.0%) are restricted to narrower ranges: West–Central Palaearctic (8 spp.), Western Palaearctic (19 spp.), European (84 spp.), Mediterranean (55 spp.), and Macaronesian (5 spp.) (Jedlička et al. 2024).

In superspecific taxa, the proportion of strictly Palaearctic units is lower. Of the 22 species groups, three (13.6%) are restricted to the Palaearctic, another five (22.7%) have their core areas in the Palaearctic but with minor extensions into the Oriental region, and one (4.5%), the *ruficorne* species group, ranges across the Palaearctic, Oriental, and Afrotropical regions. Another 13 (59.1%) species groups have a Holarctic distribution, two of which also reach the Oriental region. Of the 14 subgenera, three (21.4%) are Palaearctic, two (14.3%) extend into the Oriental region, and five (35.7%) are Holarctic, in one case with an extension into the Neotropics; the remaining subgenera have broader distributions. At the generic level, Holarctic taxa prevail: seven of the nine genera are Holarctic, *Urosimulium* is West Palaearctic, and *Simulium* is cosmopolitan.

## Conclusions

The present revision provides a critically consolidated overview of the European blackfly fauna. At the continental scale, the blackfly fauna is comparatively well documented.

The key comprehensive sources of data are Inventory (Adler 2025), Fauna Europaea (Crosskey 2017), and Catalogue of Palaearctic Diptera (Rubtsov and Yankovsky 1988), listed in decreasing order of importance. Together, these sources reflect the development of faunistic knowledge over the past half century, yet none of them provides a complete account of accepted species with verified European records. For 112 species, additional published records from individual countries were retrieved, which partly extend the lists presented in the KCS. In total, published sources document the occurrence of 268 extant, non-synonymised species-level taxa, forming the Raw Data Pool.

The raw data pool contained several poorly supported taxa. To establish a more reliable basis, a core species set (CSS) was created by excluding informally named taxa (two morphoforms and five cytoforms) and two nomina nuda, while including two revalidated species formerly treated as synonyms of *S. ruficorne*. The resulting CSS comprises 261 species.

Not all published records included in the CSS can be accepted without reservation, owing to unresolved taxonomic and nomenclatural problems, questionable zoogeographic occurrence in Europe, or possible misidentifications. To provide a transparent framework, the CSS was divided into two non-overlapping subsets:

The List of accepted species comprises 233 species with reliably documented occurrence in Europe *sensu lato* as currently recognised. Acceptance of a species at the continental level does not, however, constitute confirmation of doubtful occurrence in individual countries; such cases must be verified separately at the local level.

The List of questionable species consists of 28 species that cannot currently be regarded as confirmed members of the European fauna, either because of unresolved taxonomic or nomenclatural issues, or because of doubts about their identification supported by zoogeographic evidence. Species included in this list should not be reported from Europe or used in faunistic syntheses unless they are reassigned to the List of accepted species following prior evaluation and reliable confirmation of either their nomenclatural status or their occurrence in Europe.

This revision thus provides a transparent framework for the current knowledge of European blackflies, highlighting both the well-documented diversity and the fields where further taxonomic and faunistic research is required.

## Additional information

### Artificial Intelligence (AI) use

The authors accept full responsibility for the content of the manuscript, including the disclosure of any use of AI.

No AI tools were used in the preparation of this manuscript.

### Author ORCIDs

L. Jedlička https://orcid.org/0000-0002-5078-1170

M. Kúdela https://orcid.org/0000-0002-3604-6030

T. Kúdelová https://orcid.org/0000-0002-4553-1142

### Data availability

All of the data that support the findings of this study are available in the main text or Supplementary Information.

## Supplementary materials

Supplementary material 1 List of accepted European blackfly species (Diptera: Simuliidae) (LAS) Data type docx Explanation note The table presents a tabulated list of simuliid species whose occurrence in Europe is considered accepted and undisputed (LAS). https://binary.pensoft.net/file/1770623This dataset is made available under the Open Database License (http://opendatacommons.org/licenses/odbl/1.0/). The Open Database License (ODbL) is a license agreement intended to allow users to freely share, modify, and use this Dataset while maintaining this same freedom for others, provided that the original source and author(s) are credited. Ladislav Jedlička, Matúš Kúdela, Tatiana Kúdelová

Supplementary material 2 List of questionable blackfly species (Diptera: Simuliidae) Data type docx Explanation note The table presents a list of questionable blackfly species whose presence in Europe has been reported but remains uncertain, either due to unclear species identity or identification or to doubts about the reliability of the records. https://binary.pensoft.net/file/1770624This dataset is made available under the Open Database License (http://opendatacommons.org/licenses/odbl/1.0/). The Open Database License (ODbL) is a license agreement intended to allow users to freely share, modify, and use this Dataset while maintaining this same freedom for others, provided that the original source and author(s) are credited. Ladislav Jedlička, Matúš Kúdela, Tatiana Kúdelová
