# Supplementary material for: European blackflies: accepted and questionable species (Diptera, Simuliidae)
Source: Zookeys. 2026 Sep 14;1291:251–81. doi: 10.3897/zookeys.1291.196803 (PMC13594990; doi:10.3897/zookeys.1291.196803)
Supplement: Supplementary file 1 [file zookeys-1291-251_article-196803__-s001.docx]

**Main article: Jedlička L, Kúdela M, Kúdelová T (2026) European black flies: accepted and questionable species (Diptera: Simuliidae).**

**Supplementary file 1. List of accepted European blackfly species (Diptera: Simuliidae) (LAS)**

Table S1 presents a tabulated list of simuliid species whose occurrence in Europe is considered accepted and undisputed (LAS). The table is organised into three sections:

The **Systematic section** (green): includes supraspecific systematic categories (from tribe to species group) and valid species names. Taxa are arranged alphabetically within each higher-level category. Each entry is separated from the preceding taxon of the same rank by a horizontal line spanning the remaining width of the table. Synonyms are excluded here, as these are fully documented in the Inventory (Adler 2025).

The **Distribution section** (blue): presents species occurrence across geographical units, following the Inventory (Adler 2025).

The **Supplementary section** (no fill): contains additional species occurrence records in geographical units not covered by the Inventory (Adler 2025), followed by source citations and/or our own notes on the species' distribution data. All cited sources are provided in the References section at the end of the supplement.

(For ease of use, the supplement is formatted without top or bottom margins; scrolling is recommended for viewing.)

**Table S1.** List of accepted European blackfly species (LAS)

| tribe | | |  | species | **distribution (Adler 2025)** | **addenda et corrigenda** |
| --- | --- | --- | --- | --- | --- | --- |
|  | genus | |  |  |  |  |
|  |  | subgenus | |  |  |  |
|  |  |  | species group |  |  |  |
| **Prosimuliini** | | | |  |  |  |
|  | ***Helodon*** | | |  |  |  |
|  |  | ***Helodon*** | | ***ferrugineus*** (Wahlberg, 1844) | Sweden; Estonia, Finland, Norway, Russia (CR, NR), Siberia (WS) | Russia (KM): Sharkov et al. (1984), Yankovsky (2006), Aibulatov and Baryshev (2016) |
|  | ***Prosimulium*** | | |  |  |  |
|  |  |  | ***hirtipes*** | ***albense***  Rivosecchi, 1961 | Italy (incl. Sicily); Algeria |  |
|  |  |  |  | ***anatoliense***  Adler & Şirin, 2015 | Turkey; Russia (Caucasus) |  |
|  |  |  |  | ***calabrum*** Rivosecchi, 1966 | Italy |  |
|  |  |  |  | ***fulvipes***  (Edwards, 1921) | Bulgaria; Czech Republic, Hungary, Italy, Slovakia, Turkey | Hungary: wrong record based on a misinterpretation of the location (Zwick 1974), cf. Jedlička (1984, 1996), Papp (2001), however, occurrence cannot be ruled out despite the absence of direct evidence |
|  |  |  |  | ***gigas***  Rubtsov, 1956 | Russia (Caucasus) |  |
|  |  |  |  | ***hirtipes***  (Fries, 1824) | Sweden; Andorra, Austria, Belgium, Bosnia and Herzegovina, Britain (En, Sc), China (He, IM, Li), Czech Republic, Finland, France, Germany, Hungary, Ireland, Italy, Latvia, Luxembourg, Norway, Poland, Romania, Russia (CR, NR), Siberia (ES, WS), Slovakia, Slovenia, Spain, Switzerland, Ukraine, Greece (?), Netherlands (?). [Not North America (misidentified)] | Bulgaria: Kovachev (1976, 1985a, b, 2000)  Italy: presence is doubtful (Ciadamidaro and Mancini 2022)  Macedonia: Kovachev et al. (1999)  Montenegro: Đuknić et al. (2026)  Russia (KM): Sharkov et al. (1984), Sukhomlin and Zinchenko (2013a), Kaplich et al. (2012)  Siberia (FE): Patrusheva (1982), Aibulatov (2015) |
|  |  |  |  | ***italicum*** Rivosecchi, 1967 | Italy (incl. Sicily) |  |
|  |  |  |  | ***latimucro*** (Enderlein, 1925) | France (incl. Corsica); Austria, Bosnia and Herzegovina, Britain (En, Sc, Wa), Bulgaria, Czech Republic, Germany, Ireland, Italy, Montenegro, Morocco, Norway, Poland, Portugal, Romania, Slovakia, Spain, Switzerland, Ukraine, [Sweden’ error] | Russia (KM, NR): Ostroushko et al. (2007) ex Sharkov et al. (1984) as *inflatum.* These records could also be interpreted as *macropyga*, since according to Adler (2025), *latimucro* sensu Rubtsov & Carlsson (1965)—not Enderlein—should be considered a synonym of this species. However, Rubtsov and Carlsson (1965) listed only *inflatum*, not *latimucro*. |
|  |  |  |  | ***luganicum***  Rubtsov, 1956 | Russia (St Petersburg CR, KM); Finland, Latvia, Norway, Poland, Sweden, Romania (?) |  |
|  |  |  |  | ***petrosum***  Rubtsov, 1955 | Azerbaijan; Armenia, Bulgaria, Georgia, Lebanon, Russia (Caucasus), Ukraine (Crimea) | Ukraine (incl.? Crimea): The species was recorded from Crimea by Rubtsov (1956) and Panchenko (1999, 2003) as *nigritum*, and by Usova (1987) as *petrosum nigritum*. It was later listed from continental Ukraine by Kaplich et al. (2012), Sukhomlin and Zinchenko (2013a, b, 2016, 2018), and Teplyuk (2019), but is absent from the species list of continental Ukraine compiled by Panchenko (2016). Verification of its occurrence in continental Ukraine is advisable, as the material used (larvae, pupae, females) may represent misidentified specimens of *latimucro*. |
|  |  |  |  | ***rachiliense*** Djafarov, 1954 | Azerbaijan; Armenia, Bulgaria, Georgia, Greece (Rhodes), Morocco, Romania, Russia (Caucasus), Turkey |  |
|  |  |  |  | ***rufipes***  (Meigen, 1830) | Germany; Andorra, Austria, Bosnia and Herzegovina, Bulgaria, Czech Republic, France, Greece, Hungary, Italy (incl. Sicily), Latvia, Luxembourg, Montenegro, Poland, Romania, Serbia, Slovakia, Slovenia, Spain, Switzerland, Turkey, Ukraine, Algeria(?), [’Morocco’ error] | Ukraine (incl. Crimea): Panchenko (2000) |
|  |  |  |  | ***tomosvaryi*** (Enderlein, 1921) | Germany; Andorra, Austria, Armenia, Azerbaijan, Belgium, Bosnia and Herzegovina, Britain (En, Sc), Bulgaria, Croatia, Czech Republic, France, Georgia, Greece, Hungary, Ireland, Italy (incl. Sicily), Luxembourg, Morocco, Poland, Portugal, Romania, Russia (Caucasus), Serbia, Slovakia, Slovenia, Spain, Switzerland, Turkey, Ukraine | Montenegro: Đuknić et al. (2026) |
|  |  |  | ***macropyga*** | ***macropyga*** (Lundström, 1911) | Russia (Karelia KM); Finland, Norway, Kazakhstan, Russia (NR), Siberia (ES, WS), Sweden | Kyrgyzstan: Aibulatov (2015)  Mongolia: Aibulatov (2015)  Siberia (FE): Aibulatov (2015) |
|  |  |  |  | ***nenetz***  Yankovsky, 2011 | Russia (Arkhangelsk NR) |  |
|  |  |  |  | ***ursinum***  (Edwards, 1935) (complex) | Norway (Bear Island = Björnöya, mainland); Canada (BC, Lab, Nun incl. Baffin and Southampton Islands, NWT, Que, Yukon), Finland, Greenland, Iceland, Russia (KM), Sweden, USA (Alaska) | Siberia (ES): Aibulatov et al. 2024 |
|  | ***Twinnia*** | | | ***hydroides***  (Novák, 1956) | Slovakia; Austria, Czech Republic, Germany, Italy, Poland, Switzerland, Ukraine | Romania: Jedlička (2019) re-interpreting location of material deposited in ZIN (Yankovsky 2010) |
|  | ***Urosimulium*** | | | ***aculeatum*** (Rivosecchi, 1963) | Italy (Sicily, Sardinia); Balearic Islands (Majorca) |  |
|  |  |  |  | ***faurei***  (Bernard, Grenier & Bailly-Choumara, 1972) | Morocco; Algeria, Spain | Balearic Islands: maybe misinterpretation, López-Peńa and Jiménez-Peydró (2017) listed the species from the Balearic Islands citing González (1990, 1997) and González et al. (2002), the latter study citing Crosskey and Crosskey (2000) only gives the species from **E**[spagna] without **b**[alearic Islands] |
|  |  |  |  | ***juccii*** Contini, 1966 | Italy (Sardinia); Algeria (?), Tunisia (?) | Spain: Rivosecchi et al. (2007) |
| **Simuliini** | | | |  |  |  |
|  | ***Cnephia*** | | | ***eremites***  Shewell, 1952 | Canada (Northwest Territories incl. Victoria Island, Man, Nun incl. Southampton Island); Finland, Siberia (ES, FE), Sweden, USA (Alaska) | Russia (NR): Ostroushko et al. (2007 as *arborescens*)  Siberia (WS - Yamal): Patrusheva (1974 as *arborensis*) |
|  |  |  |  | ***pallipes***  (Fries, 1824) | Norway; Finland, Lithuania, Russia (CR, NR), Sweden, Ukraine, Siberia (?) | Russia (KM): Sharkov et al. (1984); Aibulatov (2015)  Siberia (WS, ES, FE): Aibulatov (2015) |
|  |  |  |  | ***toptchievi*** Yankovsky, 1996 | Ukraine |  |
|  | ***Greniera*** | | |  |  |  |
|  |  |  | ***fabri*** | **brachiata**  (Rubtsov, 1961) | Russia (Karelia KM) |  |
|  |  |  |  | **fabri**  Doby & David, 1959 | France; Algeria, Italy (incl. Sardinia), Morocco, Spain |  |
|  |  |  |  | ***ivanovae*** (Ivashchenko, 1970) | Russia (Ivanovo CR, Yakutskaya ES); Finland | Russia (NR): Ostroushko et al. (2007) ex Shubina (1986) ex Ostroushko and Beľtyukova (1983)  Siberia (ES - Yakutskaya): Adler (2025) |
|  |  |  |  | ***zverevae***  Rubtsov, 1964 | Russia (Komi NR) |  |
|  | ***Metacnephia*** | | | ***amphora***  Ladle & Bass, 1975 | Britain (England); France |  |
|  |  |  |  | ***bilineata***  (Rubtsov, 1940) | Siberia (Tyumen WS), Canada (Alb, BC, Man, Nun incl. Baffin Island, NWT, Que, Yukon), China (Xi), Finland, Kazakhstan, Norway, Russia (CR, NR), Sweden, USA (Alaska) | Russia (KM): Sharkov et al. (1984), Aibulatov and Baryshev (2016),  Siberia (ES, FE): Patrusheva (1982) |
|  |  |  |  | ***blanci***  (Grenier & Theodorides, 1953) | Morocco; Algeria, France, Italy (Sicily), Macedonia, Portugal, Romania, Serbia, Spain, Tunisia |  |
|  |  |  |  | ***breevi***  (Rubtsov, 1956) | Russia (Solovets Island NR) |  |
|  |  |  |  | ***danubica***  (Rubtsov, 1956) | ’Danube River’; Romania, ’Yugoslavia’ | ’Yugoslavia’, =Serbia: Rubtsov (1956 – Bosman, Sokolovac) |
|  |  |  |  | ***lyra***  (Lundström, 1911) | Finland; Germany, Norway, Russia (CR, KM, NR), Siberia (ES, WS), Sweden. [Other records in error] |  |
|  |  |  |  | ***nigra***  (Rubtsov, 1940) | Azerbaijan (Nakhichevan); Georgia, Romania, Russia (Caucasus), Turkey, Turkmenistan | Turkey (incl. Turkish Thrace): Şirin et al. (2015) |
|  |  |  |  | ***nuragica*** Rivosecchi, Raastad & Contini, 1975 | Italy (Sardinia); Portugal, Spain, Morocco (?) |  |
|  |  |  |  | ***sardoa***  (Rivosecchi & Contini, 1965) | Italy (Sardinia) |  |
|  |  |  |  | ***tredecimata*** (Edwards, 1920) | [Unknown: not ’England’]; Finland, Norway, Russia (KM), Sweden. [Other records in error] | Russia (NR): Rubtsov (1966), Sharkov et al. (1984) |
|  |  |  |  | ***uzunovi***  Kovachev, 1985 | Bulgaria |  |
|  | ***Simulium*** | | |  |  |  |
|  |  | ***Boophthora*** | | ***erythrocephalum*** (De Geer, 1776) | [Sweden]; Armenia, Austria, Belarus, Belgium, Britain (En, Sc, Wa), China (Xi), Croatia, Czech Republic, Denmark, Estonia, Finland, France, Germany, Greece, Hungary, Ireland, Italy (incl. Sardinia), Kazakhstan, Latvia, Lithuania, Moldova, Netherlands, Norway, Poland, Portugal, Romania, Russia (CR, KM, NR, SR), Serbia, Siberia (ES, FE, WS), Slovakia, Slovenia, Spain, Switzerland, Turkey, Ukraine, Tunisia (?), Mongolia (?) | Albania: Đuknić et al. (2026)  Bosnia and Herzegovina: Đuknić et al. (2026)  Bulgaria: Russev et al. (1976), Kovachev (1979a, b)  Montenegro: Đuknić et al. (2026) |
|  |  | ***Boreosimulium*** | |  |  |  |
|  |  |  | ***annulus*** | ***annae***  (Rubtsov, 1956) | Russia (St Petersburg CR); Sweden (?) |  |
|  |  |  |  | ***annulus*** (Lundström, 1911) | Finland; Canada (Alb, Lab, Man, NB, Nfld, Nun, NWT, Ont, Que, Sask), Norway, Russia (CR, KM), Sweden, USA (Mic, NY, Wis), Mongolia (?), Siberia (ES, WS) (?) | Russia (NR): Sharkov et al. (1984) as *elburnum*, Ostroushko et al. (2007) as *elburnum* ex Rubtsov (1962) and Gabova (1967) |
|  |  |  |  | ***olonicum*** (Usova, 1961) | Russia (Karelia KM); Norway, Sweden (?) |  |
|  |  |  | ***baffinense*** | ***annuliforme*** (Rubtsov, 1962) | Russia (Murmansk KM) | Russia (NR): Sharkov et al. (1984) as *latifrons* sensu Rubtsov (1956) citing other authors |
|  |  |  |  | ***arctium***  (Rubtsov, 1956) | Russia (Murmansk KM); Norway | Russia (NR): Sharkov et al. (1984), Ostroushko et al (2007)  Siberia (WS): Rubtsov (1956 Ural-Vojkar),  Siberia (ES): Patrusheva (1982 Taymyr) |
|  |  |  |  | ***baffinense***  Twinn, 1936 | Canada (Nunavut: Baffin Island and mainland, Alb, BC, Man, NWT, Que, Yukon); Finland, Norway, Russia (KM), Siberia (ES, FE, WS), Sweden, USA (Alaska, Utah) | Russia (NR): Shubina (1986) cit. Ostroushko and Beľtyukova (1983), Ostroushko et al. (2007) cit. Shubina (1986)  Russia (CR - Perm´skaya oblasť): Aibulatov (2014) cit. Sharkov et al. (1984, as *Lundstromia baffinensis* taken from other sources), but Perm´skaya oblasť is not listed there. |
|  |  |  |  | ***crassum***  (Rubtsov, 1956) | Russia (Karelia KM); Finland, Norway, Siberia (WS), Sweden | Russia (NR): Rubtsov and Yankovsky (1988), Ostroushko et al. (2007) ex Gabova (1976)  Siberia (ES): Patrusheva (1982) |
|  |  | ***Byssodon*** | |  |  |  |
|  |  |  | ***meridionale*** | ***maculatum*** (Meigen, 1804) | Germany; Belarus, China (He, IM, Xi), Italy, Kazakhstan, Lithuania, Moldova, Mongolia, Poland, Romania, Russia (CR, NR), Serbia, Siberia (ES, FE, WS), Ukraine, USA (Alaska). [Old records for other countries (notably Britain, France, and Spain) erroneous, applying mainly to species of subgenus *Wilhelmia*] | Russia (KM): Sharkov et al. (1984) |
|  |  | ***Eusimulium*** | | ***angustipes*** Edwards, 1915 | Britain (England, CI, Wa); Algeria, Armenia, Austria, Belarus, Bosnia and Herzegovina, China (Hebei), Croatia, Czech Republic, Denmark, Finland, France, Germany, Hungary, Ireland, Italy, Kazakhstan, Latvia, Lebanon, Luxembourg, Macedonia, Moldova, Mongolia, Morocco, Netherlands, Norway, Poland, Portugal, Romania, Russia (CR, NR, SR), Serbia, Slovakia, Spain, Sweden, Switzerland, Tunisia, Turkey, Ukraine | Belgium: Lock (2018)  Bulgaria: Kovachev (1976), Kovachev (2000) as *securiforme*  Russia (KM): Sharkov et al. (1984) as *latizonum*, Aibulatov and Baryshev (2016)  Siberia (WS-Altai): Petrozhitskaya and Rodkina (2009a, b) |
|  |  |  |  | ***argentipile*** (Rubtsov, 1962) | Russia (St Petersburg CR); Sweden (?) |  |
|  |  |  |  | **aureum**  Fries, 1824 | Sweden; Austria, Belarus, Belgium, Britain (En, Sc, Wa), China (He, Sh), Czech Republic, Denmark, Estonia, Finland, France, Germany, Iceland, Ireland, Italy, Latvia, Lithuania, Moldova, Netherlands, Norway, Poland, Portugal, Russia (CR, NR, SR), Siberia (WS), Slovakia, Spain, Sweden, Switzerland, Ukraine, Uzbekistan, Armenia (?). Bulgaria (?), Greece (?), India(?), Japan (?), Kazakhstan (?), Pakistan (?), Romania (?). Russia (Caucasus)? | Armenia: Andrianov et al. (2015)  Bulgaria: Kovachev (1976, 2000)  Macedonia: Kovachev et al (1999)  Russia (KM): Sharkov et al. (1984)  Russia (Caucasus): Budaeva et al. (2016), Aibulatov et al. (2021)  Serbia: Đuknić et al. (2026)  Ukraine (incl. Crimea): Panchenko (2013) |
|  |  |  |  | ***azorense***  (Carlsson, 1963) | Azores (Flores, Faial, Santa Maria, São Jorge, São Miguel, Terceira) |  |
|  |  |  |  | ***flexibranchium*** Crosskey, 2001 [*in* Crosskey & Malicky] | Greece (Rhodes, Crete, Kithira, Naxos) |  |
|  |  |  |  | ***guimari***  Becker, 1908 | Canary Islands (Tenerife, Gomera, Gran Canaria, La Palma) |  |
|  |  |  |  | ***krymense***  (Rubtsov, 1956) | Ukraine (Crimea); Bulgaria, Greece, Romania. [? = *nigrofusipes*] |  |
|  |  |  |  | ***maritimum*** (Rubtsov, 1956) | Russia (Krasnodar SR, Caucasus); Armenia, Austria, Azerbaijan, Bulgaria, Georgia, Italy, Ukraine (Crimea), China (Li) (?) |  |
|  |  |  |  | ***mellah***  Giudicelli & Bouzidi, 2000 | Morocco; Algeria; Spain |  |
|  |  |  |  | ***petricolum*** (Rivosecchi, 1963) | Italy (incl. Elba, Sardinia, Sicily); Algeria, Armenia, Austria, Bosnia and Herzegovina, Britain (En), Cyprus, Czech Republic, France (incl. Corsica), Germany, Greece, (incl. Lésbos, Páros, Rhodes), Ireland, Libya, Madeira, Morocco, Poland, Portugal, Serbia, Spain, Turkey, Russia (? *krymense*) | Bulgaria: Kovachev (1976) as *latizonum* sensu Knoz  Greece (incl. Crete): Adler et al. (2026)  Turkey (incl. Turkish Thrace): Şirin et al. (2015) |
|  |  |  |  | ***rubzovianum*** (Sherban, 1961) | Serbia; Algeria, Andorra, Austria, Balearic Islands (Majorca, Minorca), Bosnia and Herzegovina, Britain (En, Sc, Wa), Croatia, Cyprus, Czech Republic, Finland, France (incl. Corsica), Germany, Greece (incl. Corfu, Crete, Lésbos, Páros, Rhodes), Hungary, Iran, Ireland, Israel, Italy (incl. Sardinia, Sicily), Jordan, Libya, Macedonia, Malta, Moldova, Montenegro, Morocco, Netherlands, Norway, Portugal, Romania, Serbia, Slovakia, Slovenia, Spain, Sweden, Tunisia, Turkey, Ukraine (Crimea) | Bulgaria: Kovachev (1976, 1985a, b, 2000 as *latinum, serbicum*), Kovachev and Uzunov (1986)  Turkey (incl. Turkish Thrace): Şirin et al. (2015)  Ukraine (incl. Crimea): Teplyuk and Sukhomlin (2018) as *velutinum* |
|  |  |  |  | ***silvaticum*** (Rubtsov, 1962) | Russia (St Petersburg CR, SR); Italy, Romania, Serbia, Sweden (?) |  |
|  |  |  |  | ***velutinum***  (Santos Abreu, 1922) | Canary Islands (La Palma, Gomera, Tenerife); Algeria, Morocco | Canary Islands (Gran Canaria): Seitz (2025) |
|  |  | ***Hellichiella*** | |  |  |  |
|  |  |  | ***congareenarum*** | ***dogieli***  (Rubtsov, 1956) | Russia (Karelia KM, NR); Belarus, Canada (NWT), Finland, Lithuania, Norway, Siberia (WS), Sweden |  |
|  |  |  |  | ***latipes***  (Meigen, 1804) | Germany; Austria, Belgium, Britain (En, Sc), Denmark, Estonia, Finland, France, Hungary, Ireland, Latvia, Netherlands, Russia (CR, KM), Siberia (WS), Spain, Sweden, Switzerland, Ukraine, Tunisia (?) [’Libya’ error] | Russia (NR): Sharkov et al. (1984) as *yerburyi*, Aibulatov (2013)  Slovakia: Krčmárik et al. (2026) |
|  |  |  |  | ***meigeni***  (Rubtsov & Carlsson, 1965) | Russia (Murmansk KM); China (Li), Mongolia, Norway, Siberia (?). [‘Britain’ error] | Russia (CR): Aibulatov (2009, 2013),  Russia (NR): Sharkov et al. (1984), Aibulatov (2013)  Siberia (ES): Aibulatov (2014) |
|  |  |  |  | ***rivi***  (Ivashchenko, 1970) | Russia (Ivanovo CR) |  |
|  |  |  |  | ***saccai***  (Rivosecchi, 1967) | Italy |  |
|  |  |  |  | ***sedecimfistulata*** Rubtsov, 1963 | Poland; Germany | Ukraine: Yankovsky (2005) |
|  |  |  |  | ***tsheburovae*** (Rubtsov, 1956) | Russia (Murmansk KM); Norway, Sweden | Russia (NR): Sharkov et al. (1984),  Aibulatov (2013) |
|  |  |  |  | ***usovae***  (Golini, 1987) | Norway; Canada (Nun), Finland, Sweden |  |
|  |  | ***Montisimulium*** | | ***alizadei***  (Djafarov, 1954) | Azerbaijan; Armenia, China (Si), Georgia, Kazakhstan, Russia (Caucasus), Turkey |  |
|  |  |  |  | ***montium***  Rubtsov, 1947 | Tajikistan; Kazakhstan, Kyrgyzstan, Pakistan, Russia (Caucasus) |  |
|  |  | ***Nevermannia*** | |  |  |  |
|  |  |  | ***ruficorne*** | ***angustitarse*** (Lundström, 1911) | Finland; Algeria, Austria, Belarus, Bosnia and Herzegovina, Britain (En, Wa), China (Yu), Croatia, Czech Republic, Denmark, Estonia, France, Georgia, Germany, Greece, Hungary, Ireland, Italy, Kyrgyzstan, Latvia, Lithuania, Luxembourg, Macedonia, Moldova, Montenegro, Morocco, Netherlands, Norway, Poland, Portugal, Romania, Russia (CR, NR, SR), Serbia, Siberia (ES, WS), Slovakia, Slovenia, Spain, Sweden, Switzerland, Turkey, Ukraine | Bulgaria: Kovachev (1976, 2000)  Russia (KM): Sharkov et al. (1984)  Russia (Caucasus): Aibulatov et al. (2021)  Ukraine (incl. Crimea): Panchenko (2013) |
|  |  |  |  | ***annulipes***  Becker, 1908 (cytoform C of ruficorne Macquart, 1838 | *ruficorne* - CYTOFORM C: Canary Islands (Tenerife) | CYTOFORM C of *S. ruficorne* Macquart, 1838: Canary Islands (Tenerife)  We follow the opinion of Cherairia and Adler (2018) and accept cytoforms C as *S. annulipes* |
|  |  |  |  | ***beckeri***  Roubaud, 1906 (cytoform A1/A2 of ruficorne Macquart, 1838 | *ruficorne* - CYTOFORM A1: Algeria, Balearic Islands (Majorca), Egypt  *ruficorne* - CYTOFORM A2; Algeria, Canary Islands (Fuerteventura, Gomera, La Palma), Madeira | CYTOFORM A1 and A2 of *S. ruficorne* Macquart, 1838  Greece (Crete), Israel, Israel/Syria: Adler et al. (2026) as cytoform A1  We follow the opinion of Cherairia and Adler (2018) and accept cytoforms A1 and A2 as *S. beckeri.* |
|  |  |  |  | ***crassicaulum*** (Rubtsov, 1955) | Uzbekistan; Iran, Russia (Caucasus) |  |
|  |  |  |  | ***delizhanense*** (Rubtsov, 1955) | Armenia; Azerbaijan, Georgia; Russia (Caucasus) | Bulgaria (?): Russev et al. 1976 as *Ch.* d*elizhanse* citing unpublished paper by Kovachev  Moldova: Vasiliev (2023) |
|  |  |  |  | ***fuscipes***  (Fries, 1824) | Sweden; Hungary, Norway, Russia (KM) | Hungary to be omitted: based on Rubtsov´s (1967) record of *Prosimulium fuscipes* (von Roser, 1840) later (Rubtsov and Yankovsky 1988) as synonym of *P*. *tomosvaryi* (Enderlein, 1921), cf. Papp (2001) |
|  |  |  |  | ***ibleum***  (Rivosecchi, 1966) | Italy (Sicily, mainland); Algeria, Cyprus, Greece (Crete), Lebanon, Libya, Morocco, Tunisia, Turkey, Spain (?) | Italy (Sicily): Ciadamidaro and Mancini (2022 - Sicily only) |
|  |  |  |  | ***lundstromi*** (Enderlein, 1921) | Britain (England); Algeria, Austria, Belarus, Belgium, Bulgaria, China (Be), Croatia, Czech Republic, Denmark, Estonia, Finland, France, Germany, Hungary, Ireland, Italy, Kazakhstan, Latvia, Lebanon, Lithuania, Moldova, Morocco, Netherlands, Norway, Poland, Portugal, Russia (CR, NR, SR), Serbia, Slovakia, Spain, Sweden, Switzerland, Turkey, Ukraine | Bosnia and Herzegovina: Krek et al. (1976)  Greece: Đuknić et al. (2026)  Macedonia: Đuknić et al. (2026)  Russia (KM): Sharkov et al. (1984 as *latigonium*+*kerteszi*)  Siberia (WS - Altai): Petrozhitskaya and Rodkina (2009a, b) as *latigonia*  Ukraine (incl. Crimea): Panchenko (1999) as *latigonia* |
|  |  |  |  | ***pinhaoi***  Santos Grácio, 1985 | Portugal |  |
|  |  |  |  | ***volhynicum***  (Usova & Sukhomlin, 1990) | Ukraine | Moldova: Vasiliev and Sukhomlin (2022)  Ukraine (incl. Crimea): Panchenko (2013) |
|  |  |  | ***vernum*** | ***angustatum*** (Rubtsov, 1956) | Ukraine (Crimea); China (Li), Czech Republic, Poland, Romania, Russia (Caucasus, SR), Serbia, Slovakia; | Bulgaria: Russev et al. (1976)  Ukraine (incl. Crimea): Panchenko (2005 – continental Ukraine) |
|  |  |  |  | ***arminii***  Seitz & Adler, 2017 | Germany; Austria |  |
|  |  |  |  | ***armoricanum***  Doby & David, 1961 | France; Britain (En, Sc, Wa), Ireland, Portugal, Spain. [? = *florae*] |  |
|  |  |  |  | ***australe***  (Rubtsov, 1955) | Armenia; Azerbaijan, Georgia, Russia (SR) |  |
|  |  |  |  | ***bavaricum***  Seitz & Adler, 2009 | Germany; Austria, Italy |  |
|  |  |  |  | ***beltukovae*** (Rubtsov, 1956) | Russia (Permskaya CR, NR, SR); Andorra, Austria, Czech Republic, Finland, France, Germany, Italy, Norway, Poland, Serbia, Siberia (ES, WS), Slovakia, Slovenia, Sweden, Ukraine, Kazakhstan (?) | Bulgaria: Kovachev (1976, 2000) as *carpathica*  Bosnia and Herzegovina: Krek et al. (1976) as *carpathica*  Russia (KM): Sharkov et al. (1984)  Russia (Caucasus): Aibulatov et al. (2021) |
|  |  |  |  | ***berchtesgadense*** Seitz, Adler & Forster, 2015 | Germany |  |
|  |  |  |  | ***bertrandi***  Grenier & Dorier, 1959 | France (incl. Corsica); Austria, Bosnia and Herzegovina, Bulgaria, Czech Republic, Germany, Italy, Montenegro, Poland, Russia (CR, SR), Slovakia, Spain, Switzerland, Ukraine | Macedonia: Kovachev et al (1999) |
|  |  |  |  | ***bicorne*** Dorogostaisky, Rubtsov & Vlasenko, 1935 | Siberia (Irkutskaya ES, FE, WS); Canada (NWT, Yukon), China (He, IM, Jilin), Finland, Latvia, Mongolia, Norway, Poland, Russia (KM, NR), Sweden, USA (Alaska), Pakistan (?) | Russia (CR): Beľtyukova (1962), Aibulatov (2009) as *cornifera* |
|  |  |  |  | ***brevidens***  (Rubtsov, 1956) | Ukraine (Crimea); Algeria, Andorra, Austria, Bosnia and Herzegovina, Czech Republic, France, Germany, Italy, Lithuania, Montenegro, Morocco, Poland, Serbia, Slovakia, Spain, Turkey | Bulgaria: Kovachev (1976,1985a, 2000)  Ukraine (incl. Crimea): Panchenko (2005) |
|  |  |  |  | ***carthusiense*** Grenier & Dorier, 1959 | France (incl. Corsica); Andorra, Austria, Czech Republic, Germany, Italy, Morocco, Poland, Slovakia, Spain, Switzerland, Turkey, Ukraine | Bulgaria: Russev et al. (1976), Kovachev (1979a, b, 1985b, 2000) |
|  |  |  |  | ***chodakovi*** (Panchenko, 1998) | Ukraine (Crimea) |  |
|  |  |  |  | ***codreanui***  (Sherban, 1958) | Romania; Austria, Bosnia and Herzegovina, Bulgaria, Czech Republic, Poland, Serbia, Slovakia, Turkey, Ukraine |  |
|  |  |  |  | ***costatum*** Friederichs, 1920 | Germany; Algeria, Austria, Belgium, Bosnia and Herzegovina, Britain (En, Sc), Bulgaria, Croatia, Czech Republic, Denmark, France, Hungary, Italy, Latvia, Luxembourg, Moldova, Montenegro, Morocco, Netherlands, Norway, Poland, Romania, Russia (SR), Slovakia, Slovenia, Spain, Sweden, Switzerland, Turkey, Ukraine. [Argentina’ error, Edwards, 1931] | Serbia: Đuknić et al. (2026) |
|  |  |  |  | ***crenobium***  (Knoz, 1961) | Czech Republic; Austria, Bosnia and Herzegovina, Germany, Hungary, Poland, Romania, Serbia, Slovakia, Slovenia, Switzerland, Ukraine. [’Italy’ error] | Bulgaria: Kovachev (1976, 2000) |
|  |  |  |  | ***cryophilum*** (Rubtsov, 1959) (complex) | Russia (St Petersburg CR, NR, SR); Algeria, Andorra, Armenia, Austria, Balearic Islands (Majorca), Belgium, Bosnia and Herzegovina, Britain (En, Sc, Wa), Bulgaria, Croatia, Cyprus, Czech Republic, Denmark, Estonia, Finland, France (incl. Corsica), Germany, Greece (incl. Chios, Crete, Naxos, Lésbos), Ireland, Italy (incl. Sardinia, Sicily), Latvia, Lebanon, Luxembourg, Montenegro, Morocco, Netherlands, Norway, Poland, Portugal, Scotland, Serbia, Slovakia, Slovenia, Spain, Sweden, Switzerland, Tunisia, Turkey, Ukraine | Macedonia: Đuknić et al. (2026)  Russia (KM): Aibulatov and Baryshev (2016) |
|  |  |  |  | ***curvans***  (Rubtsov & Carlsson, 1965) | Siberia (Irkutskaya ES, FE, WS); Finland, Latvia, Mongolia, Norway, Romania, Russia (CR, KM), Sweden | Bulgaria: Kovachev (1969, 2000) needs revision  Russia (NR): Shubina (1986), Beľtyukova and Shubina (1977), Ostroushko et al. (2007) |
|  |  |  |  | ***dendrofilum*** (Patrusheva, 1962) | Siberia (Tomskaya WS, ES, FE); Belarus, Canada (Nun), Finland, Sweden, USA (Alaska) |  |
|  |  |  |  | ***djafarovi***  (Rubtsov, 1962) | Azerbaijan; Armenia, Georgia, Kazakhstan, Romania, Russia (SR), Turkey | Russia (Caucasus): Aibulatov et al. (2021) |
|  |  |  |  | ***dolomitense*** (Rivosecchi, 1971) | Italy |  |
|  |  |  |  | ***dunfellense***  Davies, 1966 | Britain (England, Sc); Belgium, Ireland |  |
|  |  |  |  | ***elatum***  (Rubtsov, 1955) | Azerbaijan; Georgia, Russia (Caucasus) |  |
|  |  |  |  | ***florae***  (Djafarov, 1954) | Azerbaijan; Georgia, Romania, Russia (Caucasus), Turkey |  |
|  |  |  |  | ***fontinale*** Radzivilovskaya, 1948 | Siberia (Maritime FE incl. Sakhalin); Canada (Alb, BC, NWT, Yukon), Finland, Japan (Hon), Mongolia, USA (Alaska) | Russia (KM): Sharkov et al. (1984)  Russia (NR): Sharkov et al. (1984) + as *longipile* (?), Shubina (1986) ex Ostroushko and Beľtyukova (1983), Ostroushko et al. (2007) as *pugetense* ex Shubina (1986)  Russia (CR): Beľtyukova (1977)  Siberia (WS): Patrusheva (1982) as *pugetense*, Petrozhitskaya and Rodkina, (2007) as *pugetense* |
|  |  |  |  | ***fontium***  (Rubtsov, 1955) | Russia (Caucasus); Armenia, Azerbaijan, Georgia, Ukraine. [? = *costatum*] | Ukraine (Crimea): Panchenko (1999) |
|  |  |  |  | ***fucense*** (Rivosecchi, 1962) | Italy |  |
|  |  |  |  | ***gejgelense*** (Djafarov, 1954) | Azerbaijan; China (Si), Georgia, Russia (Caucasus) | Ukraine (incl. Crimea): listed as Crimean-Caucasian chorotype by Panchenko (1999, 2003) but Sukhomlin and Zinchenko (2016) considered Crimean material as misidentification (*brevidens*) and the species as unconfirmed from Ukraine; the status of Carpathian record is unclear (Sukhomlin and Zinchenko 2013a) |
|  |  |  |  | ***gomphocorne*** (Rubtsov, 1964) | Russia (Caucasus) |  |
|  |  |  |  | ***hasekei***  Seitz, Adler & Remschak, 2021 | Austria |  |
|  |  |  |  | ***ichnusae*** Rivosecchi & Contini, 1994 | Italy (Sardinia), Algeria |  |
|  |  |  |  | ***juxtacrenobium*** Bass & Brockhouse, 1990 | Britain (England); Finland, Ireland, Sweden, Netherlands (?) |  |
|  |  |  |  | ***karajimae*** (Panchenko, 2003) | Ukraine (Crimea) |  |
|  |  |  |  | ***kuznetzovi***  Rubtsov, 1940 | Russia (Murmansk KM) | Russia (CR): Aibulatov (2013) |
|  |  |  |  | ***lidiae***  (Semushin & Usova, 1983) | Ukraine | Russia (CR): Budaeva and Khitsova (2010) |
|  |  |  |  | ***marsicanum*** (Rivosecchi, 1962) | Italy (incl. Sicily) |  |
|  |  |  |  | ***murvanidzei*** (Rubtsov, 1955) | Georgia; Armenia. Russia (Caucasus) |  |
|  |  |  |  | ***naturale***  Davies, 1966 | Britain (England, Sc, Wa); Denmark, Estonia, Ireland, Lithuania, Spain, Germany(?), Norway (?) |  |
|  |  |  |  | ***oligotuberculatum*** (Knoz, 1965) | Slovakia; Andorra, Austria, Czech Republic, France, Germany, Italy, Poland, Spain | Ukraine: Teplyuk and Sukhomlin (2018) |
|  |  |  |  | ***quasidecolletum*** Crosskey, 1988 [1987] | France; Austria, Germany, Italy, Spain, Switzerland |  |
|  |  |  |  | ***silvestre***  (Rubtsov, 1956) | Siberia (Irkutskaya ES, WS); Canada (Alb, BC, Lab, Man, NB, Nfld, Nun, NWT, Ont, PEI, Que incl. Magdalen Islands, Sask, Yukon), China (Si, Yu), Finland, Mongolia, Norway, Russia (KM), Sweden, Ukraine, USA (Alaska, Cal, Col, Idaho, Maine, Mic, NH, NJ, NY, Ore, Pen, Was, Wyo) | Russia (CR): Rubtsov (1956), Beľtyukova (1977), Aibulatov (2009)  Russia (NR): Sharkov et al. (1984), Shubina (1986) ex Ostroushko and Beľtyukova (1983) |
|  |  |  |  | ***tauricum***  (Rubtsov, 1956) | Ukraine (Crimea) | Ukraine (incl. Crimea): reported from Carpathians by Usova and Panchenko (1973), and as part of Carpatho-Crimean mountain group (i.e. continental Ukraine + Crimea) by Panchenko (2003), further also by Sukhomlin & Zinchenko (2016), but missing in species list from continental Ukraine by Panchenko (2016) |
|  |  |  |  | ***timondavidi*** Giudicelli, 1961 [1960] | France (Corsica) |  |
|  |  |  |  | ***urbanum***  Davies, 1966 | Britain (England); Austria, Belgium, Denmark, Germany, Ireland, Luxembourg, Spain, Sweden, Netherlands (?) |  |
|  |  |  |  | ***vernum***  Macquart, 1826 | France; Andorra, Armenia, Austria, Azerbaijan, Belarus, Belgium, Bosnia and Herzegovina, Britain (En, Sc, Wa), China (He, IM, Jilin, Li), Croatia, Czech Republic, Denmark, Estonia, Finland, Germany, Georgia, Hungary, Iceland, Ireland, Italy (incl. Sicily), Kazakhstan, Latvia, Liechtenstein, Lithuania, Moldova, Mongolia, Morocco, Netherlands, Norway, Poland, Portugal, Romania, Russia (CR, KM, NR, SR), Serbia, Siberia (FE, WS), Slovakia, Slovenia, Spain, Sweden, Switzerland, Tunisia, Turkey, Ukraine. [Not North America (misidentified)] | Bulgaria: Kovachev (1976, 1979a, b, 1985b, 2000)  Montenegro: Đuknić et al. (2026)  Russia (Caucasus): Patrusheva (1982), Khitsova and Budaeva (2014), Aibulatov et al. (2021)  Siberia (ES): Patrusheva (1982) |
|  |  |  | | ***zakhariense*** (Rubtsov, 1955) | Armenia; Azerbaijan, Georgia; | Moldova: Vasiliev (2023) |
|  |  | ***Obuchovia*** | | ***adornatum*** (Rubtsov, 1956) | Russia (Caucasus) |  |
|  |  |  |  | ***auricoma***  Meigen, 1818 | Austria; Andorra, Bulgaria, Croatia, Cyprus, Czech Republic, France (incl. Corsica), Germany, Greece (incl. Crete, Ikaría, Lésbos, Naxos, Rhodes, Samos), Italy (incl. Sardinia, Sicily), Kazakhstan, Lebanon, Morocco, Poland, Portugal, Romania, Serbia, Slovakia, Spain, Switzerland, Turkey, Ukraine | Turkey (incl. Turkish Thrace: Şirin et al. 2015) |
|  |  |  |  | ***brevifile***  (Rubtsov, 1956 | Ukraine (Crimea); Italy (incl. Sicily, Sardinia) | Ukraine (incl. Crimea): Usova and Panchenko (1973), Panchenko (2005), Sukhomlin and Zinchenko (2016) |
|  |  |  |  | ***continii***  (Rivosecchi & Cardinali, 1975) | Italy (Sardinia) |  |
|  |  |  |  | ***galloprovinciale*** Giudicelli, 1963 [1962] | France; Algeria, Italy, Morocco, Spain; Turkey |  |
|  |  |  |  | ***ibericum***  Crosskey & Santos Grácio, 1985 | Portugal; Spain |  |
|  |  |  |  | ***karasuae*** (Panchenko, 1998) | Ukraine (Crimea) |  |
|  |  |  |  | ***popowae***  Rubtsov, 1940 | Armenia; Azerbaijan, Bulgaria; Georgia, Russia (Caucasus) |  |
|  |  |  |  | ***segusinum*** (Couvert, 1968) | Italy |  |
|  |  | ***Psilozia*** | | ***vittatum***  Zetterstedt, 1838 | Greenland; Canada (Alb, BC incl. Queen Charlotte Islands, Man, Nfld, Nun, NWT, PEI, Ont, Que incl. Magdalen Islands, Sask, Yukon), Faeroe Islands, Iceland, Mexico (FD, Ta), USA (Alaska, Ari, Ark, Cal, Col, Idaho, Iowa, Maine, Mar, Mic, Min, Misr, Mon, Neb, Nev, NH, NM, NY, Ohio, Ore, Pen, SC, Utah, Ver, Vir, Was, Wis, WV, Wyo) |  |
|  |  | ***Rubzovia*** | | ***joanae***  Seitz, Zwick and Adler, 2012 | Madeira |  |
|  |  |  |  | ***lamachi***  Doby and David, 1960 | France; Germany, Morocco, Spain | Canary Islands (Las Palmas) absent: (López-Peńa and Jiménez-Peydró 2017) – error in site interpretation (Clergue-Gazeau and Vinçon 1990, Vinçon and Clergue-Gazeau 1993, Crosskey et al. 1999) |
|  |  |  |  | ***paraloutetense*** Crosskey, 1988 | Canary Islands (Gran Canaria, Gomera) |  |
|  |  | ***Schoenbaueria*** | | ***giganteum***  Rubtsov, 1940 | Siberia (WS; ES); Canada (Nun), Russia (NR) |  |
|  |  |  |  | ***ivashchenkoi*** (Yankovsky, 1996) | Russia (Ivanovo CR) |  |
|  |  |  |  | ***nigrum***  (Meigen, 1804) | Germany; Belarus, China (Xi), Lithuania, Poland, Romania, Russia (CR, SR), Ukraine, Uzbekistan | Russia (NR): Sharkov et al. (1984), Aibulatov (2013) |
|  |  |  |  | ***patrushevae*** (Ivashchenko, 1978) | Russia (Ivanovo CR) |  |
|  |  |  |  | ***pusillum***  Fries, 1824 | Finland; Belarus, Czech Republic, Estonia, Latvia, Lithuania, Mongolia, Norway, Poland, Russia (CR, KM), Siberia (ES, FE, WS), Sweden, Ukraine | Russia (NR): Sharkov et al. (1984), Shubina (1986) ex Ostroushko and Beľtyukova (1983), Aibulatov (2014) |
|  |  |  |  | ***raastadi***  (Usova & Reva, 2000) | Ukraine |  |
|  |  |  |  | ***rangiferinum*** (Rubtsov, 1956) | Russia (Archangel NR); Siberia (ES, WS) |  |
|  |  |  |  | ***rubzovium*** (Ivashchenko, 1978) | Russia (Ivanovo CR |  |
|  |  |  |  | ***subpusillum*** Rubtsov, 1940 | Siberia (Irkutskaya ES [neotype], WS); Canada (Nun, NWT), Finland, Kazakhstan, Russia (CR, NR), Sweden, USA (Alaska); Ukraine (?) | Russia (KM): Usova (1961), Sharkov et al. (1984)  Siberia (FE): Patrusheva (1982)  Ukraine: Kaplich et al. (2011), Panchenko (2016) |
|  |  |  |  | ***suchomlinae*** (Usova & Reva, 1995) | Ukraine |  |
|  |  | ***Simulium*** | |  |  |  |
|  |  |  | ***argenteostriatum*** | ***argenteostriatum*** Strobl, 1898 | Bosnia and Herzegovina; Algeria, Andorra, Austria, Bulgaria, Czech Republic, France, Germany, Greece, Italy, Montenegro, Poland, Romania, Serbia, Slovakia, Slovenia, Spain, Switzerland, Tunisia, Turkey, Ukraine | Albania: Đuknić et al. (2026)  Belarus: Dovnar (2017) as *schönbaueri* (?)  Macedonia: Kovachev et al (1999) |
|  |  |  |  | ***hispaniola***  Grenier and Bertrand, 1954 | Spain; Algeria, France, Italy, Portugal, Turkey |  |
|  |  |  | ***bezzii*** | ***bezzii***  (Corti, 1914) (complex) | Italy (incl. Sardinia, Sicily); Algeria, Andorra, Armenia, Austria, Azerbaijan, Bosnia and Herzegovina, Bulgaria, Croatia, Cyprus, France (incl. Corsica), Georgia, Germany, Greece (incl. Crete, Lésbos, Rhodes), Iran, Iraq, Israel, Lebanon, Macedonia, Montenegro, Morocco, Poland, Romania, Russia (Caucasus), Serbia, Spain, Switzerland, Turkey, Ukraine, Uzbekistan | Balearic Islands: López-Peña and Jiménez-Peydró (2017) citing Gonzáles (1990, 1997); however, González et al. (2002) consider these earlier records not confirmed.  Slovenia: Đuknić et al. (2026)  Turkey (incl. Turkish Thrace): Şirin et al. (2015)  Ukraine (incl. Crimea): Panchenko (1999, 2003) as *kondici* |
|  |  |  | ***bukovskii*** | ***bukovskii***  Rubtsov, 1940 | Ukraine (Crimea); Armenia, Bulgaria, Romania, Russia (SR), Turkey | Russia (Caucasus): Khitsova and Budaeva (2014), Budaeva et al. (2016)  Turkey (incl. Turkish Thrace: Şirin et al. (2015) |
|  |  |  |  | ***degrangei***  Dorier & Grenier, 1960 | France; Austria, Bosnia and Herzegovina, Bulgaria, Georgia, Germany, Greece, Italy, Montenegro, Serbia, Slovakia, Switzerland, Turkey, Ukraine | Albania: Đuknić et al. (2026)  Armenia: Andrianov et al. (2015)  Slovenia: Đuknić et al. (2026) |
|  |  |  |  | ***vigintifile*** (Dinulescu, 1966) | Romania |  |
|  |  |  | ***malyschevi*** | ***cholodkovskii*** Rubtsov, 1940 [1939] | Siberia (Buryatskaya ES, FE, WS); China (He, Jilin), Kazakhstan, Mongolia, Russia (CR) |  |
|  |  |  |  | ***decimatum*** Dorogostaisky, Rubtsov & Vlasenko, 1935 | Siberia (Baikal ES, FE, WS); China (He), Kazakhstan, Mongolia, Russia (CR, KM) | Russia (NR): Beľtyukova and Shubina (1977), Sharkov et al. (1984), Shubina (1986, 2007), Ostroushko et al. (2007) |
|  |  |  |  | ***gabovae***  (Rubtsov, 1966) | Russia (Komi NR) |  |
|  |  |  |  | ***ibariense*** Zivkovitch & Grenier, 1959 | Serbia; Austria, Bulgaria, Czech Republic, Macedonia, Poland, Switzerland, Ukraine |  |
|  |  |  | ***noelleri*** | ***dolini***  Usova & Sukhomlin, 1989 | Ukraine; Belarus | Moldova: Vasiliev and Sukhomlin (2022)  Russia (CR): Vasilevich and Kaplich (2016) |
|  |  |  |  | ***noelleri***  Friederichs, 1920 (complex) | Germany; Armenia, Austria, Belarus, Belgium, Bosnia and Herzegovina, Britain (CI, En, Sc, Wa), Canada (Man, Nun, NWT, Yukon), China (IM, Li), Czech Republic, Denmark, Estonia, Finland, France, Hungary, Ireland, Italy, Kazakhstan, Latvia, Lithuania, Luxembourg, Mongolia, Netherlands, Norway, Poland, Romania, Russia (CR, KM, NR, SR), Serbia, Siberia (ES, WS, FE), Slovakia, Slovenia, Sweden, Switzerland, Turkey, Ukraine, USA (Alaska) | Bulgaria: Kovachev (1985b as *argyreatum*, 2000)  Greece: Đuknić et al. (2026)  Montenegro: Đuknić et al. (2026)  Russia (Caucasus): Aibulatov et al. (2021)  Turkey (incl. Turkish Thrace): Şirin et al. (2015)  Ukraine (incl. Crimea): Panchenko (1999) |
|  |  |  |  | ***palustre***  Rubtsov, 1956 | Siberia (Irkutskaya ES, FE, WS); China (IM), Ukraine | Russia (CR): Aibulatov (2013) |
|  |  |  |  | ***tshuni***  (Yankovsky, 2006) | Russia (Murmansk KM) |  |
|  |  |  | ***ornatum*** | ***baracorne***  Smart, 1944 | Serbia; Bulgaria, Greece, Moldova, Romania, Russia (Caucasus), Slovakia, Turkey, Ukraine (incl. Crimea), Uzbekistan |  |
|  |  |  |  | ***bronchiale*** (Rubtsov, 1962) | Russia (St Petersburg CR) | Russia (KM): Aibulatov and Baryshev (2016) |
|  |  |  |  | ***croaticum*** (Baranov, 1937) | Croatia; Germany (?) |  |
|  |  |  |  | ***fontanum*** Terteryan, 1952 | Armenia; Azerbaijan, Iran, Romania, Russia (SR), Turkey | Russia (Caucasus): Budaeva and Khitsova (2016)  Ukraine: Sukhomlin et al. (2008), Sukhomlin and Zinchenko (2008, 2013b) |
|  |  |  |  | ***frigidum***  Rubtsov, 1940 | Siberia (ES, WS); Belarus, Finland, Lithuania, Norway, Poland, Romania, Russia (CR, NR), Sweden, Ukraine | Bulgaria (?): Russev et al. (1976), Kovachev (1979a)  Russia (KM): Aibulatov and Baryshev (2016), Sharkov et al. (1984) |
|  |  |  |  | ***fuscum***  (Rubtsov, 1963) | Russia (St Petersburg CR, NR) | Russia (KM): Aibulatov and Baryshev (2016) |
|  |  |  |  | ***intermedium*** Roubaud, 1906 | Canary Islands (Tenerife, Gomera, Gran Canaria, La Palma); Algeria, Andorra, Austria, Balearic Islands (Majorca, Minorca), Belarus, Belgium, Britain (En, Sc, Wa), Bulgaria, Denmark, Finland, France (incl. Corsica), Georgia, Greece, Germany, Ireland, Italy (incl. Sardinia, Sicily), Lithuania, Luxembourg, Madeira, Malta, Moldova, Montenegro, Morocco, Netherlands, Norway, Poland, Portugal, Romania, Serbia, Slovenia, Spain, Sweden, Tunisia, Ukraine. [’Lebanon’, as *nitidifrons*, error] | Russia (KM): Aibulatov and Baryshev (2016)  Russia (NR): Sharkov et al. (1984) as *nitidifrons*? |
|  |  |  |  | ***kiritshenkoi*** Rubtsov, 1940 | Iran; Armenia, Azerbaijan, Bulgaria, Cyprus, Georgia, Iraq, Pakistan, Romania, Russia (Caucasus), Tajikistan, Turkey, Ukraine | Macedonia: Kovachev et al (1999) as *caucasica*  Turkey (incl. Turkish Thrace): Şirin et al. (2015) |
|  |  |  |  | ***ornatum***  Meigen, 1818 (complex) | Germany; Algeria, Andorra, Austria, Belarus, Belgium, Bosnia and Herzegovina, Britain (CI, En, Sc, Wa), Bulgaria, China (Gu, Jilin, Li, Yu), Croatia, Czech Republic, Denmark, Estonia, Finland, France, Georgia, Greece (incl. Crete, Rhodes), Hungary, Ireland, Israel, Italy (incl. Sardinia, Sicily), Jordan, Kazakhstan, Latvia, Lebanon, Liechtenstein, Lithuania, Luxembourg, Macedonia, Moldova, Mongolia, Montenegro, Morocco, Netherlands, Norway, Poland, Portugal, Romania, Russia (CR, KM, NR, SR), Serbia, Siberia (ES, FE, WS), Slovakia, Slovenia, Spain, Sweden, Switzerland, Tunisia, Turkey, Turkmenistan, Ukraine, Uzbekistan; [Kyrgyzstan error] | Albania: Đuknić et al. (2026)  Russia (Caucasus): Antipina et al. (1979), Aibulatov et al. (2021)  Turkey (incl. Turkish Thrace): Şirin et al. (2015) |
|  |  |  |  | ***pontinum*** Rivosecchi, 1960 | Italy |  |
|  |  |  |  | ***rotundatum*** (Rubtsov, 1956) | Russia (Murmansk KM, CR); Bulgaria, Norway, Poland, Romania, Sweden, Ukraine |  |
|  |  |  |  | ***savici***  (Baranov, 1937) | Serbia; Croatia |  |
|  |  |  |  | ***trifasciatum***  Curtis, 1839 | Britain (England, CI); Algeria, Andorra, Austria, Belgium, Bulgaria, Croatia, Czech Republic, Denmark, France, Germany, Greece, Hungary, Ireland, Italy, Lithuania, Morocco, Netherlands, Poland, Portugal, Slovakia, Slovenia, Spain, Switzerland, Turkey, Ukraine | Albania: Đuknić et al. (2026)  Macedonia: Kovachev et al. (1999) as *spinosa*  Montenegro: Đuknić et al. (2026)  Serbia: Đuknić et al. (2026)  Ukraine (incl. Crimea): Panchenko (1999, 2000) |
|  |  |  | ***pavlovskii*** | ***murmanum*** Enderlein, 1935 | Russia (KM, CR); Canada (Alb, BC, Lab, Man, NB, Nfld, NS, NWT, Ont, Que, Sask, Yukon), Finland, Norway, Siberia (ES, FE incl. Sakhalin, WS), Sweden, Ukraine**,** USA Alaska, Maine, Mas, Mic, Min, NH, NY, Ver, Wis) | Mongolia: Yadamsuren et al. (2020)  Russia (NR): Sharkov et al. (1984) as *tenuimanus* Rubtsov (?)  Ukraine: not listed by Panchenko (2016); reported from the Ukrainian Carpathians by Sukhomlin and Zinchenko (2016) as a correction of *S. pavlovskii* misidentified by Usova (1987) |
|  |  |  | ***reptans*** | ***banaticum*** Dinulescu, 1966 | Romania |  |
|  |  |  |  | ***colombaschense*** (Scopoli, 1780) (complex) | Serbia; Austria, Bosnia and Herzegovina, Bulgaria, Croatia, Germany, Greece, Hungary, Italy, Macedonia, Romania, Slovakia, France (?). [’Lithuania’ error] | Ukraine: Teplyuk and Sukhomlin (2018), Teplyuk and Teplyuk (2019) |
|  |  |  |  | ***liriense***  Rivosecchi, 1961 | Italy |  |
|  |  |  |  | ***reptans***  (Linnaeus, 1758) (complex) | Sweden /’Lapland’; Austria, Belarus, Belgium, Bosnia and Herzegovina, Britain (En, Sc, Wa), Bulgaria, China (He, Xi), Croatia, Czech Republic, Denmark, Finland, France, Germany, Greece, Hungary, Ireland, Italy, Kazakhstan, Latvia, Liechtenstein, Lithuania, Luxembourg, Macedonia, Montenegro, Netherlands, Norway, Poland, Portugal, Romania, Russia (CR, KM, NR incl. Novaya Zemlya, SR, Serbia, Siberia (ES, WS), Slovakia, Spain, Switzerland, Tunisia, Turkey, Ukraine, Uzbekistan;  ‘reptans A’ Kúdela, Jedlička, Brúderová & Bernotiene, 2012 (molecular form) Britain (Wa), Russia (KM), Sweden  ‘*reptans* B’ Kúdela, Jedlička, Brúderová & Bernotiene,  2012 (molecular form) Britain (Wa), Croatia, Hungary, Kazakhstan, Latvia, Lithuania, Macedonia, Siberia (ES, WS), Slovakia, Sweden | Russia (Caucasus): Antipina et al. (1979) as *galeratum* (?)  Slovenia: Đuknić et al. (2026)  Turkey (incl. Turkish Thrace): Şirin et al. (2015) |
|  |  |  |  | ***reptantoides*** Carlsson, 1962 (complex) | [unspecified European]; Bosnia and Herzegovina, Britain (En, Wa), Bulgaria, Croatia, Germany, Montenegro, Serbia, Slovakia  ‘*reptantoides* A’ Bosnia and Herzegovina, Britain (En, Wa), Bulgaria, Croatia, Germany, Hungary, Serbia, Slovakia  ‘*reptantoides* B’ Albania, Bosnia and Herzegovina, Britain (En, Wa), Bulgaria, Germany, Hungary, Montenegro, Serbia, Slovakia | Austria: Seitz (2019)  Slovenia: Đuknić et al. (2026)  Ukraine: Teplyuk and Sukhomlin (2018) |
|  |  |  |  | ***tarnogradskii*** Rubtsov, 1940 | Russia (Caucasus); Armenia, Azerbaijan, China (Xi), Georgia, Uzbekistan |  |
|  |  |  |  | ***voilense*** Sherban, 1960 | Romania; Croatia, Hungary, Italy, Serbia, Slovakia, Ukraine |  |
|  |  |  |  | ***zetterstedti*** Carlsson, 1962 | Sweden |  |
|  |  |  | ***slossonae*** | ***transiens***  Rubtsov, 1940 | Siberia (Irkutskaya ES, FE, WS); Canada (Alb, BC, Man, NWT, Sask, Yukon), China (IM), Finland, Latvia, Mongolia, Poland, Russia (CR, KM, NR), Sweden, USA (Alaska) |  |
|  |  |  | ***tuberosum*** | ***annulitarse*** Zetterstedt, 1838 | Sweden; Canada (Nun), Finland, Russia (CR, KM), Siberia (FE) |  |
|  |  |  |  | ***corpulentum*** Rubtsov, 1956 | Russia (Permskaya CR, SR) |  |
|  |  |  |  | ***gusevi*** Rubtsov, 1976 | Russia (Kuybyshev CR) |  |
|  |  |  |  | ***janzeni***  Enderlein, 1922 | Austria; Latvia, Romania | Bulgaria: Kovachev (1985b as *tuberosum*?)  Russia (KM): Sharkov et al. (1984), Aibulatov and Baryshev (2016)  Russia (NR): Sharkov et al. (1984)  Russia (CR): Aibulatov (2009)  In accordance with the opinion of Zwick (1995), we consider the records published in the Inventory (Adler 2025) under the name *tuberosum* with a question mark to be assigned to the name *janzeni* as follows:  Andorra (?), Belarus (?), Belgium (?), Bosnia and Herzegovina (?), Britain [not *tuberosum* s. str.] (En, Sc, Was), Bulgaria (?), Croatia (?), Czech Republic (?), France (?), Germany [not tuberosum s. str.], Ireland (?), Lithuania (?), Poland (?), Portugal (?), Romania (?), Slovakia (?), Slovenia (?), Spain (?), Switzerland (?), Turkey (?), Ukraine (?). |
|  |  |  |  | ***polare***  Rubtsov, 1940 | Siberia (Taymyrsky WS, ES); Russia (NR, SR) | Russia (KM): Yankovsky (2006) |
|  |  |  |  | ***splendidum*** Rubtsov, 1940 | Siberia (Irkutskaya ES, FE, WS); China (Manchuria’), Russia (KM) |  |
|  |  |  |  | ***subtile***  Rubtsov, 1956 | Russia (Caucasus); Italy, Romania |  |
|  |  |  |  | ***tuberosum*** (Lundström, 1911) | Finland; Canada (Alb, BC, Lab, Man, NB, Nfld, Nun, NWT, Ont, PEI, Que, Sask, Yukon), Norway, Russia (CR, KM, NR), Siberia (ES, FE, WS), Sweden, USA (Ala, Alaska, Ark, Col, Flo, Geo, Ind, Ken, Maine, Mic, Min, Misp, Misr, Mon, NC, NH, NJ, NM, NY, Ohio, Okl, Pen, SC, Ten, Vir, Wis, WV), [‘Mexico’ error] | Only unquestioned records from the Inventory are included (Adler 2025); questioned records are attributed to *S. janzeni*. |
|  |  |  |  | ***tumulosum*** Rubtsov, 1956 | Siberia (Irkutskaya ES, FE, WS); China (Jilin), Finland, Mongolia, Norway, Russia (NR), Sweden | Russia (KM): Sharkov et al. (1984)  Russia (CR): Aibulatov (2013) |
|  |  |  |  | ***vulgare*** Dorogostaisky, Rubtsov & Vlasenko, 1935 | Siberia (Irkutskaya ES, FE, WS); Austria, Canada (Alb, Nun, NWT, Yukon), China (He), Czech Republic, Finland, Germany, Kazakhstan, Latvia, Mongolia, Norway, Romania, Russia (CR, NR, SR), Serbia, Slovakia, Sweden, Ukraine, USA (Alaska), Uzbekistan | Bosnia and Herzegovina: Đuknić et al. (2026)  Russia (KM): Sharkov et al. (1984) |
|  |  |  | ***variegatum*** | ***argyreatum*** Meigen, 1838 | Germany; Andorra, Austria, Bosnia and Herzegovina, Britain (En, Sc, Wa), Bulgaria, Croatia, Czech Republic, Finland, France, Greece, Hungary, Ireland, Italy, Luxembourg, Macedonia, Montenegro, Norway, Poland, Portugal, Romania, Russia (SR), Serbia, Slovakia, Slovenia, Spain, Sweden, Switzerland, Turkey, Ukraine, Kazakhstan (?) | Russia (KM): Aibulatov and Baryshev (2016),  Russia (NR): Shubina (1986) ex Ostroushko and Beľtyukova (1983)  Russia (CR): Beľtyukova (1977)  Russia (Caucasus): Antipina et al. (1979)  Siberia (WS): Patrusheva (1974)  Turkey (incl. Turkish Thrace): Şirin et al. (2015)  Older published records from northern and central Russia and Siberia are likely misidentifications; species identities remain uncertain. |
|  |  |  |  | ***debacli***  Terteryan, 1952 | Armenia; Azerbaijan, Bulgaria, Georgia**,** Romania, Russia (Caucasus), Turkey |  |
|  |  |  |  | ***laplandicum*** (Chubareva & Yankovsky, 1992) | Russia (Murmansk KM) |  |
|  |  |  |  | ***maximum***  (Knoz, 1961) | Czech Republic; Austria, Bosnia and Herzegovina, Bulgaria, France, Germany, Italy, Poland, Romania, Serbia, Slovakia, Spain, Switzerland, Ukraine | Greece: Đuknić et al. (2026)  Montenegro: Đuknić et al. (2026) |
|  |  |  |  | ***monticola*** Friederichs, 1920 | Germany; Algeria, Andorra, Azerbaijan, Austria, Belgium, Bosnia and Herzegovina, Bulgaria, Croatia, Czech Republic, Finland, France (incl. Corsica), Georgia, Italy, Luxembourg, Macedonia, Norway, Poland, Portugal, Romania, Russia (Caucasus, CR, NR, SR), Serbia, Slovakia, Slovenia, Spain, Sweden, Switzerland, Ukraine. [‘Britain’ error]; | Greece: Đuknić et al. (2026)  Montenegro: Đuknić et al. (2026)  Russia (KM): Aibulatov and Baryshev (2016), Sharkov et al. (1984) as *monticola stenostylus* |
|  |  |  |  | ***monticoloides*** (Rubtsov, 1956) | Russia (Caucasus); Bulgaria, Georgia, Romania, Turkey, Ukraine (Crimea) | Ukraine (Crimea): missing in the whole Ukraine (incl. Crimea) - Panchenko (1999, 2003, 2016), Sukhomlin and Zinchenko (2016)  Bulgarian and Romanian records need confirmation (Kúdela 2006) |
|  |  |  |  | ***schamili***  (Rubtsov, 1964) | Russia (Caucasus); Armenia, Georgia |  |
|  |  |  |  | ***sicanum*** (Rivosecchi, 1963) | Italy (Sicily) | Italy (incl. Sicily, i.e. in peninsular Italy also) Ciadamidaro and Mancini (2022) |
|  |  |  |  | ***variegatum***  Meigen, 1818 | Germany; Algeria, Andorra, Armenia, Austria, Azerbaijan, Belgium, Bosnia and Herzegovina, Britain (En, Sc, Wa), Bulgaria, Croatia, Czech Republic, France (incl. Corsica), Georgia, Greece (incl. Andros, Rhodes), Hungary, Iran, Ireland, Italy (incl. Sicily), Lebanon, Montenegro, Morocco, Poland, Portugal, Romania, Russia (Caucasus), Serbia, Slovakia, Slovenia, Spain, Sweden, Switzerland, Turkey, Ukraine, Kazakhstan (?), Pakistan (?), Uzbekistan (?).[Cyprus’ error] | Albania: Đuknić et al. (2026)  Macedonia: Kovachev et al (1999)  Turkey (incl. Turkish Thrace): Şirin et al. (2015) |
|  |  |  |  | ***xanthinum*** Edwards, 1933 | Spain; Algeria, France, Morocco |  |
|  |  |  | ***venustum*** | ***abbreviatum*** Rubtsov, 1957 | Russia (St Petersburg CR); Ukraine |  |
|  |  |  |  | ***aemulum***  Rubtsov, 1940 | Siberia (Irkutskaya ES, FE, WS); China (Li), Mongolia | Russia (KM): Sharkov et al. (1984)  Russia (NR - Archangeľsk): Sharkov et al. (1984)  Russia (CR): Aibulatov (2013) |
|  |  |  |  | ***kachvorjanae*** Usova & Zinchenko, 1991 | Ukraine |  |
|  |  |  |  | ***longipalpe*** Beltyukova, 1955 | Russia (Permskaya CR, KM, NR, SR); Belarus, China (IM), Finland, Kazakhstan, Lithuania, Norway, Siberia (ES, FE, WS), Sweden, Ukraine, Uzbekistan |  |
|  |  |  |  | ***lugense***  Yankovsky, 1996 | Russia (St Petersburg CR); Ukraine | Russia (KM): Sharkov et al. (1984) as *hibernale* |
|  |  |  |  | ***morsitans***  Edwards, 1915 | Britain (England, Sc, Wa); Austria, Belarus, Belgium, China (IM), Czech Republic, Denmark, Estonia, Finland, France, Germany, Kazakhstan, Latvia, Lithuania, Luxembourg, Mongolia, Netherlands, Norway, Poland, Romania, Russia (CR, SR), Siberia (ES, FE), Slovakia, Slovenia, Sweden, Turkey, Ukraine | Bulgaria: Russev et al. (1976), Kovachev (1985a, 2000), Kovachev and Uzunov (1986)  Russia (KM): Sharkov et al. (1984), Aibulatov and Baryshev (2016)  Russia (NR): Ostroushko et al. (2007), Sharkov et al. (1984)  Siberia (WS): Mitrokhin (1973) |
|  |  |  |  | ***paramorsitans*** Rubtsov, 1956 | Russia (St Petersburg CR, KM, NR, SR); Belarus, Bosnia and Herzegovina, Bulgaria, Czech Republic, Estonia, Finland, Germany, Hungary, Italy, Kazakhstan, Latvia, Lithuania, Mongolia, Norway, Poland, Romania, Serbia, Siberia (ES, FE, WS), Slovakia, Sweden, Ukraine | Netherlands: Mulder (2002), Knoz and Beuk (2020) |
|  |  |  |  | ***posticatum***  Meigen, 1838 | Germany; Austria, Belarus, Belgium, Britain (En), Czech Republic, Denmark, Estonia, Finland, France, Latvia, Lithuania, Luxembourg, Netherlands, Norway, Poland, Russia (CR, KM), Siberia (WS), Slovenia, Sweden, Turkey, Ukraine | Bulgaria: probably present in Bulgaria, maybe hidden under *verecundum*; all records (see Hubenov 2021) need a revision  Russia (NR): Sharkov et al. (1984) as *austeni*, Shubina (1986) ex Ostroushko and Beľtyukova (1983) as *verecundum* (?)  Historical published records under *venustum* and *verecundum* are misidentifications, and the species identities require verification. |
|  |  |  |  | ***promorsitans*** Rubtsov, 1956 | Russia (Orenburgskaya CR, SR); Belarus, China (IM), Kazakhstan, Lithuania, Poland, Siberia (ES, WS), Ukraine | Russia (KM): Sharkov et al. (1984)  Russia (NR): Aibulatov (2013), Sharkov et al. (1984) |
|  |  |  |  | ***rivosecchii***  Rubtsov, 1964 | Italy |  |
|  |  |  |  | ***rostratum*** (Lundström, 1911) | Finland; Austria, Belarus, Britain (En, Sc), Canada (Alb, BC incl. Queen Charlotte Islands, Lab, Man, Nfld, Nun, NWT, Ont, PEI, Que incl. Magdalen Islands, Sask, Yukon), Czech Republic, Denmark, Finland, Germany, Greenland, Ireland, Latvia, Lithuania, Norway, Poland, Russia (NR), Siberia (FE incl. Sakhalin, WS), Slovakia, Sweden, Ukraine, USA (Alaska, Idaho, Maine, Mic, Min, Mon, NH, NY, Ore, Was, Wis, Wyo) | Bulgaria: Kovachev (1976, 1985b, 2000) as *verecundum* (?) (see note under *posticatum*  Kazakhstan: Makatov (2008a)  Russia (CR): Aibulatov (2009, 2013), Beľtyukova (1977) as *verecundum* (?)  Rus (KM): Sharkov et al. (1984 as *verecundum* (?), Aibulatov and Baryshev (2016)  Siberia (ES): Bobrova (1977)  Historical published records under *venustum* and *verecundum* are misidentifications, and the species identities require verification. |
|  |  |  |  | ***rubtzovi***  Smart, 1945 | Siberia (Buryatskaya ES, FE, WS); Canada (NWT, Yukon), Finland, Kazakhstan, Russia (CR, SR), Sweden, Ukraine, USA (Alaska) | Mongolia: Yadamsuren et al. (2020)  Russia (KM): Sharkov et al. (1984), Aibulatov and Baryshev (2016)  Russia (NR): Sharkov et al. (1984), Ostroushko et al. (2007) ex Shubina (1986) ex Ostroushko and Beľtyukova (1983) |
|  |  |  |  | ***shevtshenkovae*** Rubtsov, 1965 | Ukraine; Belarus, Russia (CR, SR) |  |
|  |  |  |  | ***simulans***  Rubtsov, 1956 | Russia (Orenburgskaya CR, SR); Belarus, Kazakhstan, Siberia (ES, FE), Ukraine | Russia (KM): Sharkov et al. (1984) |
|  |  |  |  | ***truncatum*** (Lundström, 1911) | Finland; Belarus, Canada (Alb, BC, Lab, Nfld, Nun, NWT, Ont, Que, Sask), Lithuania, Norway, Russia (KM), Siberia (ES, FE, WS), Sweden, Ukraine, USA (Alaska, Maine, Mic, NH, NY) | Russia (NR): Sharkov et al. (1984), Ostroushko et al. (2007)  Russia (CR): Bel'tjukova and Leykina (1964), Aibulatov (2009) |
|  |  | ***Wilhelmia*** | |  |  |  |
|  |  |  | ***equinum*** | ***angustifurca*** (Rubtsov, 1956) | Russia (Caucasus); Bulgaria, China (IM), Ukraine | Ukraine present: Panchenko (2012), Sukhomlin and Zinchenko (2016, 2018) but omitted by Panchenko (2016)  Ukraine (Crimea): Panchenko (1999, 2003) as SE-Euro-Crimeo-Caucasian chorotype |
|  |  |  |  | ***balcanicum*** (Enderlein, 1924) | Bulgaria; Albania, Austria, Belarus, Bosnia and Herzegovina, Croatia, Germany, Greece, Hungary, Italy, Macedonia, Moldova, Montenegro, Poland, Romania, Russia (SR, CR), Serbia, Slovakia, Slovenia, Turkey, Ukraine (Crimea), Lithuania (?) | Bulgaria: Kovachev (1976, 1985a, b), Kovachev and Uzunov (1986)  Russia (Caucasus): Budaeva et al. (2016)  Turkey (incl. Turkish Thrace): Şirin et al. (2015)  Ukraine (incl. Crimea): Kaplich et al. (2015), Usova and Panchenko (1973) |
|  |  |  |  | ***dahestanicum*** (Rubtsov, 1962) | Russia (Dagestan); Iran; Iraq, ‘Transcaucasus’ | Moldova: Vasiliev (2022, 2025)  Russia (Caucasus): Aibulatov et al. (2021) |
|  |  |  |  | ***equinum***  (Linnaeus, 1758) (complex) | ‘Nordlandiae’ [Scandinavia]; Armenia, Austria, Azerbaijan, Belarus, Belgium, Bosnia and Herzegovina, Britain (En, Sc, Wa), Bulgaria, Czech Republic, China (Qi, Sg, Xi), Croatia, Denmark, Estonia, Finland, France, Georgia, Germany, Greece, Hungary, Ireland, Italy (incl. Sardinia), Kazakhstan, Latvia, Liechtenstein, Lithuania, Macedonia, Moldova, Montenegro, Morocco, Netherlands, Norway, Poland, Portugal, Romania, Russia (CR, KM, NR, SR), Serbia, Siberia (ES, FE, WS), Slovakia, Slovenia, Spain, Sweden, Switzerland, Turkey, Ukraine. [‘Algeria’, ‘Libya’, ‘Cyprus’ errors] | Albania: Đuknić et al. (2026)  Turkey (incl. Turkish Thrace): Şirin et al. (2015) |
|  |  |  |  | ***lineatum***  (Meigen, 1804) | Germany; Austria, Belarus, Belgium, Bosnia and Herzegovina, Britain (En, Wa), Bulgaria, Croatia, Czech Republic, Denmark, France (incl. Corsica), Greece, Hungary, Ireland, Italy (incl. Sardinia, Sicily), Latvia, Lithuania, Montenegro, Netherlands, Poland, Portugal, Romania, Russia (CR, SR), Serbia, Slovakia, Slovenia, Spain, Sweden, Switzerland, Ukraine. [‘Azores’, ‘Libya’ errors] | Albania: Đuknić et al. (2026)  Macedonia: Đuknić et al. (2026)  Kazakhstan: Makatov (2008a, b)  Ukraine (incl. Crimea): Panchenko (2000) |
|  |  |  |  | ***paraequinum***  Puri, 1933 (complex) | Pakistan; Armenia, Azerbaijan, Bulgaria, Croatia, Greece (incl. Lésbos), Hungary, Iran, Iraq, Israel, Italy, Jordan, Lebanon, Montenegro, Romania, Russia (SR), Serbia, Turkey, Ukraine (Crimea) | Russia (Caucasus): Budaeva and Khitsova (2016)  Turkey (incl. Turkish Thrace: Şirin et al. (2015)  Ukraine (incl. Crimea): from continental Ukraine reported by Teplyuk and Sukhomlin (2018) |
|  |  |  |  | ***pseudequinum*** Séguy, 1921 (complex) | Canary Islands (Gran Canaria, Gomera, Tenerife); Algeria, Armenia, Austria, Azerbaijan, Bosnia and Herzegovina, Britain (En), Bulgaria, China (Sx, Xi), Croatia, Cyprus, France, Georgia, Greece (incl. Andros, Chíos, Crete, Ikaría, Lésbos, Náxos, Rhodes), India (Kashmir, Pu), Iran, Iraq, Israel, Italy (incl. Sardinia, Sicily), Jordan, Kazakhstan, Kyrgyzstan, Lebanon, Libya, Macedonia, Moldova, Montenegro, Morocco, Pakistan, Portugal, Romania, Russia (Caucasus), Serbia, Slovakia, Slovenia, Spain, Tajikistan, Tunisia, Turkey, Turkmenistan, Ukraine (incl. Crimea), Uzbekistan, Czech Republic (?); | Albania: Đuknić et al. (2026)  Turkey (incl. Turkish Thrace): Şirin et al. (2015) |
|  |  |  |  | ***quadrifila*** Grenier, Faure & Laurent, 1957 | Morocco; Algeria, Spain |  |
|  |  |  |  | ***sergenti*** Edwards, 1923 | Algeria; Morocco, Portugal, Spain, Tunisia |  |
|  |  |  |  | ***veltistshevi*** Rubtsov, 1940 | Tajikistan; Afghanistan, Azerbaijan, China (IM, Xi), Georgia, Iran, Kazakhstan, Pakistan, Romania, Turkey, Turkmenistan, Ukraine (incl. Crimea), Uzbekistan |  |
|  | ***Stegopterna*** | | | ***duodecimata*** (Rubtsov, 1940) | Siberia (Irkutskaya ES, FE, WS); Norway | Russia (NR): Ostroushko et al. (2007) ex Sharkov et al. (1984), Aibulatov (2015)  Russia (CR): Aibulatov (2015) citing multiple sources, Rubtsov´s (1956) record based on females from southern Ural (Azyam river) needs verification |
|  |  |  |  | ***trigonium*** (Lundström, 1911) (complex) | Finland; Belarus, Canada (Nun, NWT), Denmark, Japan (Hon), Latvia, Norway, Russia (CR, KM, NR), Siberia (ES, FE, WS), Sweden, Ukraine, USA (Alaska) Netherlands (?). [‘Romania’ error] |  |

References

Mulder JL (2002) Twee nieuwe Simulidaesoorten voor Nederland. Macrofaunanieuwsmail 25: 2

Ostroushko TS, Bel’tyukova KN (1983) Preimaginaľnye stadii moshek basseyna r. Shchugor. Trudy Komi Filiala AN SSSR 57: 68–77.

Ostroushko TS, Paniukova EV, Pestov CV (2007) Dvukrylye nasekomye (Insecta: Diptera) kompleksa gnus fauny evropejskogo severo-vostoka Rossii. Trudy Komi Nauchnogo Centra UrO Rosiyskoy AN 183: 190–235.

Panchenko AA (1999) K analizu geograficheskich elementov fauny moshek (Diptera, Simuliidae) Krymskogo poluostrova. Problemy Ekologii i Okhrany Prirody Tekhnogennogo Regiona 1: 75–87.

Panchenko AA (2000) O bioraznoobrazii mukh semeystva Simuliidae (Diptera) na yuzhnom makrosklone Krymskikh gor. Visti Kharkivskogo Entomologichnogo Tovaristva 8(2): 46–49.

Panchenko AA (2003) K zoogeograficheskomu analizu fauny moshek (Diptera: Simuliidae) Ukrainy. Problemy Sovremennoy Parazitologii 2: 47–50.

Panchenko AA (2005) K faune moshek (Diptera: Simuliidae) severnogo makrosklona Ukrainskikh Karpat. Zoocenosis - 2005. In: Bioriznomattya ta Roľ Zoocenozu v Pryrodnykh i Antropogennykh Ekosystemakh, Materialy III Mizhnarodnoy Naukovoy Konferencii, Dnepropetrovsk, October 2005. Vid DNU, Dnepropetrovsk, 290–292.

Panchenko AA (2012) K faune moshek (Diptera, Simuliidae) ravninnoy Ukrainy. In: XIV S´ezd Russkogo Entomologicheskogo Obshchestva Sankt-Peterburg 27 August – 1 September 2012, Materialy s´ezda. Galanika, Sankt-Peterburg, 342.

Panchenko AA (2013) Nekotorye svedeniya o moshkakh roda Nevermannia Enderlein 1921 (Diptera: Simuliidae) v Krymu. Naukovi Zapiski Derzhavnogo Prirodoznavchogo Museyu 29: 121–128.

Panchenko AA (2016) Ob inventarizacii sovremennoy fauny moshek (Diptera: Simuliidae) materikovoy Ukrainy. In: Ovchinnikova OG, Kustov SYu, Gladun VV, Barkalov AV, Krivosheina MG, Nagalevskiy MV, Narchuk EP, Negrobov OP, Shamshev IV, Shatalkin AI, (Eds) Grootaert PX. Verossiyskiy Dipterologicheskiy Simpozium, Krasnodar, August 2016. Kubanskiy Gos.Un-t, Krasnodar, 261–266.

Papp L (2001) Simuliidae. In: Papp L (Ed.) Checklist of the Diptera of Hungary. Hungarian Natural History Museum, Budapest, 87–89.

Patrusheva VD (1974) O moshkakh (Diptera, Simuliidae) Yuzhnogo Yamala. Parazitologiya 8(2): 138–146.

Patrusheva VD (1982) Moshki Sibiri i Daľnego Vostoka (annotirovannyy katalog-spravochnik vidov). Nauka, Novosibirsk, 321 pp.

Petrozhitskaya LV, Rodkina VI (2007) Taksonomicheskiy sostav i landshaftno-biotopicheskoe raspredelenie moshek (Ditera, Simuliidae) v basseyne reki Khemchik (Zapadnaya Tuva). Parazitologiya 41: 241–52.

Petrozhitskaya LV, Rodkina VI (2009a) Relationship between blackfly (Diptera: Simuliidae) distribution in the Sema River Basin (North Altai). Acta Zoologica Lituanica, 19: 318–323. <https://doi.org/10.2478/v10043-009-0038-z>

Petrozhitskaya LV, Rodkina VI (2009b). The Spatial Distribution of Black Flies (Diptera: Simuliidae) in the Basin of the Sema Mountain River in the North Altai Mountain Region. Inland Water Biology, 2(1), 33–41. [https://doi.org/10.1134/ S1995082909010064](https://doi.org/10.1134/%20S1995082909010064)

Rivosecchi L, Addonisio M, Maiolini B (2007) I Ditteri Simulidi: Nuove chiavidicotomiche per l’dentificazione delle specie italiane con brevi note bio-tassonomiche. Quaderni del Museo Tridentino di Scienze Naturali, Trento, 2, 148 pp.

Rubtsov IA (1956) Moshki (Simuliidae). 2nd ed. Vol. 6. Fauna SSSR. Izd. AN SSSR, Moskva, 860 pp.

Rubtsov IA (1962) Moshki v pishche ryb bassejna reki Usy. In: Zvereva OS (Ed.) Ryby bassejna reki Usy i ih kormovye resursy. Izdateľstvo Akademii nauk SSSR, Moskva-Leningrad, 264-268.

Rubtsov IA (1966) Moshki (sem. Simulidae) sistemy Vashutkinykh ozer. In: Belyaev GM, Vinberg GG, Gaevskaya NC (Eds) Gidrobiologitcheskoe izutchenie i rybokhozyay-stvennoe osvoenie ozer kraynego Severa SSSR. Nauka, Moskva, 103–112.

[Rubtsov] Rubzow IA (1967) Über Die Kriebelmücken Ungarns. Annales Historico-Naturales Musei Nationalis Hungarici.Pars Zoologica 59: 303–318.

[Rubtsov] Rubtzov IA, Carlsson G (1965) On the Taxonomy of Black Flies from Scandinavia and Northern USSR. Acta Universitatis Lundensis Sectio II 18: 3–40.

[Rubtsov] Rubzow IA, Yankovsky AV (1988) Family Simuliidae. In: Soós A, Papp L (Eds) Catalogue of Palaearctic Diptera 3, Akademiai Kiadó, Budapest, 114–186.

Russev B, Kovachev S, Janewa I, Karapetkowa M, Uzunov J, Detschewa R (1976) Vertreter der bulgarischen Flussfauna als limnosaprobe Bioindikatoren. Hidrobiologia 4: 60–66.

Seitz G (2019) 4.5.9 Kriebelmücken (Simuliidae). In: Remschak C., Haseke H. Benthosuntersuchungen in Bächen, Quellen und Teichen im Rahmen des LIFE+ Projektes Ausseerland (Steirisches Salzkammergut). Mitteilungen des Naturwissen-schaftlichen Vereines für Steiermark, Graz, 119–124.

Seitz G (2025) Current state of the blackfly (Simuliidae) fauna of the Canary Islands, with new records. Graellsia 81: e751.

Sharkov AL, Lobkova ML, Usova ZV (1984) Krovososushchie komary (sem. Culicidae) i moshki (sem. Simuliidae) Evropeyskogo Severa SSSR. Kareliya, Petrozavodsk, 151 pp.

Shubina VN (1986) Gidrobiologiya Lososevoy Reki Severnogo Urala. Nauka, Leningrad, 158  pp.

Shubina VN (2007) Fauna i ekologiya donnykh bespozvonochnykh lososevykh rek Urala i Timana. DrSc Thesis, Institut biologii Komi Nauchnogo tsentra Ural’skogo otdeleniya

Adler P.H. (2025) World Blackflies (Diptera: Simuliidae): A Comprehensive Revision of the Taxonomic and Geographical Inventory [2025]. 148 pp.

<https://biomia.sites.clemson.edu/pdfs/blackflyinventory.pdf>

Adler PH, Seitz G, Kampen H, Werner D (2026) Chromosomal and distributional insights into the cytoforms of Simulium ruficorne (Diptera: Simuliidae). Zootaxa 5831 (2): 267–273. <https://doi.org/10.11646/zootaxa.5831.2.8>

Aibulatov SV (2009) Bloodsucking Dipterawns (Diptera: Ceratopogonidae, Culicidae, Simuliidae, Tabanidae) of the Kurgala Peninsula, Leningrad Province. Entomological Review 89(6): 645–58. <https://doi.org/10.1134/S0013873809060037>

Aibulatov SV (2013) Fauna moshek (Diptera: Simuliidae) Leningradskoy i Vologodskoy oblastey. In: Prokin AA, Petrov PN, Zhavoronkova OD, Tuzovskiy PV (Eds) Gidroentomogiya v Rossii i sopredeľnykh stranakh: Materialy V Vserossiyskogo simpoziuma po amfibioticheskim i vodnym nasekomym, Bor`ok (Russia), Filigran’, Yaroslavl, 10–12.

Aibulatov SV (2014) K faune moshek trib Nevermanniini i Wilhelmiini (Diptera: Simuliidae) Respubliki Sakha (Yakutia). Parazitologiya 48(6): 409–422.

Aibulatov SV (2015) A Contribution to the Fauna of Blackflies of the Subfamily Prosimuliinae (Diptera: Simuliidae) of the Sakha Republic (Yakutia). Entomological Review 95(1): 61–72. <https://doi.org/10.1134/S001387381501008X>

Aibulatov SV, Baryshev IA (2016) K faune moshek (Diptera: Simuliidae) Karelii i Murmanskoy oblasti. Problemy vodnoy entomologii Rossii i sopredel’nykh stran, 2016, 8–12.

Aibulatov SV, Budaeva IA, Palatov DM, Yakimov AV (2021) K Faune Moshek (Diptera: Simuliidae) Kabardino-Balkarii i Karachaevo-Cherkessii. In Problemy Vodnoy Entomologii Rossii i Sopredel’nykh Territoriy, 13–16.

Aibulatov SV, Przhiboro AA, Fedorov DD (2024) Fauna moshek (Diptera, Simuliidae) plato Putorana. In: XII All-Russian Dipterological Symposium. Program and abstracts. Russkoe Entomologicheskoe Obshchestvo, St.-Petersburg, 13.

Andrianov BV, Goryacheva II, Vlasov SV, Gorelova TV, Harutyunova MV, Harutyunova KV, Mayilyan KR, Zakharov IA (2015) Identifikaciya potenciaľno invazivnykh vidov chernykh moshek (Diptera: Simuliidae) Armenii na osnove analiza izmenchivosti shtrikhkodovogo gena mtDNK Cox1 i khromosomnogo polimorfizma. Genetika 51(3): 351–61. <https://doi.org/10.1134/S1022795415020039>

Antipina II, Chumakova IV, Trufanov GV (1979) K faune moshek (Simuliidae) Centraľnogo Kavkaza. Parazitologiya 13(3): 262–262. <https://doi.org/10.1080/00222937900770191>

Bel’tyukova KN (1962) K Izucheniju Moshek (Simuliidae) v Permskoi oblasti. Uchenye Zapiski Permskogo Gosudarstvennogo Universiteta imeni A.M.Gorkogo 22(4): 91–100.

Bel’tyukova KN (1977) Moshki (Diptera, Simuliidae) Yuzhnogo Urala. Voprosy arachnoentomologii: Fauna i ekologia paukov i krovososushchikh chlenistonogikh, 24–45.

Bel’tyukova KN, Leykina GA (1964) Krovososushchie Moshki (Diptera, Simuliidae) Srednego Prikam’ya. Uchenye Zapiski Permskogo Gosudarstvennogo Universiteta imeni A.M.Gorkogo 114: 35–38.

Bel’tyukova KN, Shubina VN (1977) Moshki (Diptera, Simuliidae) reki Shchugor i ikh roľ v pishche ryb. Biologicheskie Resursy Belogo Morya i Vnutrennikh Vodoemov Evropeyskogo Severa, 1977: 38–40.

Bobrova SI (1977) Roenie krovososushchikh moshek (Simuliidae) v Sibiri. Parazitologiia, 11(5): 442–445.

Budaeva IA, Khitsova LN (2010) The Structure of the Fauna and Life Cycles of Black Flies (Diptera, Simuliidae) in Streams of the Forest-Steppe in Central Russia. Entomological Review 90: 1192–1201.

Budaeva IA, Khitsova LN (2016) Faunisticheskiy obzor moshek (Diptera: Simuliidae) Kavkaza. In: X Verossiyskiy Dipterologicheskiy Simpozium, Krasnodar, 56–60.

Budaeva IA, Khitsova LN, Shapovalov MI, Saprykin MA (2016) Fauna moshek (Diptera: Simuliidae) Respubliki Adygeya. Problemy Vodnoy Entomologii Rossii i Sopredel’nykh Stran, 2016, 19–27.

Ciadamidaro S, Mancini L (2022) The new Checklist of the Italian Fauna: Simuliidae. Biogeographia - The Journal of Integrative Biogeography, 37: ucl008 <https://doi.org/10.21426/B637257479>

Cherairia M, Adler PH (2018) Genetic Variation in a Colonization Specialist, Simulium ruficorne (Diptera: Simuliidae), the World’s Most Widely Distributed Black Fly. *PLOS One* 13(10): e0205137. <https://doi.org/10.1371/journal.pone.0205137>

Crosskey RW, Crosskey ME (2000) An investigation of the blackfly fauna of Andalusia, southern Spain (Diptera: Simuliidae). Journal of Natural History 34: 895–951. <https://doi.org/10.1080/002229300299309>

Crosskey RW, Malmqvist B, Nilsson AN (1999) A review of the Palaearctic blackfly subgenus Simulium (Rubzovia) with emphasis on the discovery of the aquatic stages of S. (R.) paraloutetense, a species confined to Gran Canaria Island (Diptera: Simuliidae). Entomologica Scandinavica 29: 383–393.

Dovnar DV (2017) Krovososushchie moshki (Diptera, Simuliidae) severnoy lesorastiteľnoy podzony Belarusi. In: Yanchurovich OV (Ed.) Zoologicheskie Chteniya 2017, Sbornik statey Mezhdunarodnoy nauchno-prakticheskoy konferencii Grodno (Belarus), March 2017. GrGU im. Ya. Kupaly, Grodno, 77–78.

Đuknić J, Jovanović VM, Marinković N, Raković M, Atanacković A, Paunović M, Popović N (2026) Environmental drivers of the diversity and distribution of black flies ( Diptera:

Rossiyskoy akademii nauk, Syktyvkar.

Şirin Ü, Çalışkan H, Şahin Y (2015) Black flies (Diptera: Simuliidae) of Turkish Thrace, with a new record for Turkey. Biodiversity Data Journal 3: e4834. doi: 10.3897/BDJ.3.e4834 <https://doi.org/10.3897/BDJ.3.e4834>

Sukhomlin KB, Zinchenko OP 2008. Fauna moshok rodini Simuliidae Ukrainskikh Karpat. Naukovij Visnik Uzhgorodskogo Universitetu, Seria Biologii 23: 230–232.

Sukhomlin KB, Zinchenko OP (2013a) Osoblivosti Simuliidofauny Zakhodu Ukrainy. Naukoviy Visnik Uzhorodskogo Universitetu Seriya Biologiya 35: 104–108.

Sukhomlin K, Zinchenko O (2013b) Vpliv geografichnogo polozhennya na zabarvlennya tila ta ozmiri preimaginaľnykh faz rozvitky simuliid. Naukovyi Visnik Skhidnoevropey-skogo Natsionaľnogo Universytetu Imeni Lesi Ukrayinky Zoologiya IV Zoologiya 14: 176–180.

Sukhomlin KB, Zinchenko OP (2016) Taksonomichna rizmanitist moshok (Diptera, Simuliidae). Ukrainskiy Entomologicheskiy Zhurnal 11(1–2): 38–45.

Sukhomlin K, Zinchenko O (2018) Pro kolektsiyu moshok (Simuliidae, Diptera) kafedri zoologiyi Skhidnoevropeyskogo natsional’nogo universitetu imeni Lesi Ukrayinki. Naukoviy Visnik Skhidnoevropeys’kogo Natsionaľnogo Universytetu imeni Lesi Ukrayinki, Seriya Biologichni Nauki, II Zoologiya 4(377): 48–57.

Sukhomlin KB, Zinchenko OP, Teplyuk VS (2008) Do pitannya pro poshirennya moshok (Diptera, Simuliidae) v basseyni richki Tereblya. Visnik Prikarpats´kogo Natsionaľno-go Universitetu, seriya Biologiya 12: 75–77.

Teplyuk AM (2019) Species Composition and Biotopical Distribution of Blackflies (Diptera, Simuliidae) on the South-Western Macroslope of the Ukrainian Carpathians. Vestnik Zoologii 53(2): 113–122. <https://doi.org/10.2478/vzoo-2019-0011>

Teplyuk AM, Sukhomlin KB (2018) Prostorovo-tipologichna struktura ugrupovan’ moshok (Diptera: Simuliidae) baseynu richki Tisi v mezhakh Ukrayins’kikh Karpat. Ukrayins’kiy Entomologitchniy Zhurnal 15(2): 59–70. <https://doi.org/10.15421/281815>

Teplyuk A, Teplyuk V (2019) Ekologo-faunistichniy analiz moshok (Diptera, Simuliidae) gidrobiotsenoziv pivnichno-skhidnogo makroskhilu Ukrayins’kikh Karpat. Visnik Kiyivs’kogo Natsionaľnogo Universitetu im. Tarasa Shevchenka Biologiya 77(1): 32–38.

Usova ZV (1961) Fauna moshek Karelii i Murmanskoy oblasti (Diptera, Simuliidae). Izdateľstvo AN SSSR, Moscow-Leningrad, 286 pp.

Usova ZV (1987) Zonaľnoe raspredelenie moshek (Diptera, Simuliidae) Ukrainy. In: Vinogradova EV (Ed.) Krovososushchie Dvukrylye i ikh Kontroľ, Leningrad, 133–136.

Usova ZV, Panchenko AB (1973) O mestakh vyploda moshek (Diptera, Simuliidae) Zakarpatskoj oblasti. Parazitologiya 7: 541–544.

Vasilevich F, Kaplich VM (2016) Ecology of preimaginal stages of blood-sucking black flies in central non-chernozem zone of the Russian federation. International Journal of Research in Ayurveda and Pharmacy 7(3): 152–154. <https://doi.org/10.7897/2277-4343.074174>

Vasiliev A (2022) First evidence of Simulium dahestanicum (Rubtsov, 1962) in Europe. In: Joint ESENIAS and DIAS Scientific Conference 2022 and 11th ESENIAS Workshop Invasive alien species under conditions of global crisis 13-15 November 2022, Book of Abstracts, Demre, Antalya, 49.

Vasilie A (2023) Discoveries of non-native Araneae (Arachnida) and Simuliidae (Diptera) species in the Republic of Moldova. In: Globalisation and invasive alien species in the Black Sea and Mediterranean regions – management challenges and regional cooperation. IBER-BAS, ESENIAS, DIAS, Varna, 109.

Vasiliev AI (2025) Simulium dahestanicum (Rubtsov, 1962) (Diptera, Simuliidae) in the Republic of Moldova: New Insights into the Morphology and Separation from Closely Related Species. Entomological Review 105: 510–520. https://doi.org/10.1134/S0013873825070036

Vasiliev AI, Sukhomlin KB (2022) New records of Simulium volhynicum (Usova & Sukhomlin, 1990) and Simulium dolini Usova & Sukhomlin, 1989 (Diptera, Simuliidae) from the Republic of Moldova. In: Materialy II Mezhdunarodnoy nauchno-prakticheskoy konferentsii Proceedings of the II International scientific and practical conference, Minsk. 545–548.

Vincon G, Clergue-Gazeau M (1993) Les simulies (Diptera, Simuliidae) du sud-ouest de l’Europe: le crenal et l’epirhithral. Annales de Limnologie 29: 157–169.

Yadamsuren O, Morse JC, Hayford B, Gelhaus JK, Adler PH (2020) Macroinvertebrate community responses to land use: A trait-based approach for freshwater biomonitoring in Mongolia. Hydrobiologia 847: 1887–1902. <https://doi.org/10.1007/s10750-020-04220-2>

Yankovsky AV (2005) Moshki (Diptera: Simuliidae) rodov *Hellichiella* Rivosecchi et Cardinali, 1975 i *Boreosimulium* Rubzov et Yankovsky, 1982. Parazitologiya 39(6): 508–515.

Yankovsky AV (2006) Blackflies (Diptera, Simuliidae) of the Lapland State Biosphere Nature Reserve, with Description of a New Species of the Genus Argentisimulium Rubzov et Yankovsky, 1982. Entomological Review, 86(2): 240–246. [https://doi.org/10.1134/ S0013873806020114](https://doi.org/10.1134/%20S0013873806020114)

Yankovsky AV (2010) Black Flies of the Genera *Gymnopais* Stone, 1949 and *Twinnia* Stone et Jamnback, 1955 (Diptera: Simuliidae) from the Fund Collection of the Zoological Institute of the Russian Academy of Sciences. Proceedings of the Zoological Institute RAS 314: 149–72. <https://doi.org/10.31610/trudyzin/2010.314.2.149>

Zwick H (1974) Faunistisch-Ökologische und Taxono-mische Untersuchungen an Simuliidae (Diptera) unter Besonderer Berücksichtigung der Arten des Fulda-Gebietes. Abhandlungen Senckengbergischen Naturforschenden Gesellschaft 533: 1–116

Zwick H (1995) Contribution to the European blackfly taxa (Diptera: Simuliidae) named by Enderlein. Aquatic Insects 17: 129–173.

Simuliidae) in the Balkan Peninsula. Insect Conservation and Diversity 19: 308–317. https://doi.org/10.1111/icad.70021

Gabova EN (1967) Fauna i biologiya moshek basseyna r. Kos'yu (Intinskiy rayon Komi ASSR). Izvestiya Komi filiala Vsesoyuznogo geograficheskogo obshchestva. 2(1): 84-91.

González G (1990) Sistemática y ecología de los Simuliidae (Diptera) de los ríos de Catalunya y de otras cuencas hidrográficas españolas. Tesis de Licenciatura, Universidad de Barcelona, Barcelona, Spain.

González G, (1997) Claves para la identificación de las larvas y pupas de los Simulidos (Diptera) de la Península Ibérica. Asociación Española de Limnología, Barcelona. 77 p.

González G, Crosskey RW, Báez M. (2002) Simuliidae. In: M. Carles-Tolrá Hjorth-Andersen (Ed.) Catálogo de Los Diptera de Espaňa, Portugal y Andorra (Insecta). Monografias Sociedad Entomológica Aragones, Zaragoza, 75-77.

Hubenov Z (2021) Species composition and distribution of the Dipterans (Insecta: Diptera) in Bulgaria. Pensoft, National Museum of Natural History, Sofia, 276 pp. <https://doi.org/10.3897/ab.e68616>

Inci A, Yildirim A, Duzlu O, Onder Z, Ciloglu A, Seitz G, Adler PH (2017) Genetic Diversity and Identification of Palearctic Black Flies in the Subgenus Wilhelmia (Diptera: Simuliidae). Journal of Medical Entomology 2017: 1–7.

Jedlička L (1984) Simuliidae. In: Čepelák J (Ed) Diptera Slovenska I. Veda, Bratislava, 74-83, 242-243.

Jedlička L (1996) A synopsis of blackfly fauna of Slovakia (Diptera, Simuliidae). Acta Zoologica Universitatis Comenianae 40: 49–67.

Jedlička L (2019) Jan Amos Novák and the Story about his Twinnia Species. The Simuliid Bulletin 52: 3–14.

Kaplich VM, Sukhomlin KB, Zinchenko OP (2011). Zoogeografichnyi Analiz simuliido-fauny Polissya. Naukovyi Visnik Volynskogo Natsionaľnogo Universytetu imeni Lesi Ukrainky Zoologiya 19: 68–75.

Kaplich VM, Sukhomlin KB, Zinchenko OP (2012) Morfofunktsionaľni adaptacii imago simuliid, povyazani z osoblivostyami mists meshkaniya. Naukovyi Visnik Volynskogo Natsionaľnogo Universytetu im. Lesi Ukrainky Zoologiya 2(2): 36–41.

Kaplich VM, Sukhomlin EB, Zinchenko AP (2015) Moshki (Diptera: Simuliidae) smeshannykh lesov Evropy. Novoe znanie, Minsk, 464 pp.

Khitsova LN, Budaeva IA. (2014) Moshki (Diptera, Simuliidae) Severo-Zapadnogo Kavkaza. Izdateľskiy dom VGU, Voronezh, 184 pp.

Kovachev S (1976) Simuliidae (Diptera) ot porechiata na rekite Struma i Mesta. Hydrobiology (Sofia), 3: 67–77.

Kovachev S (1979a) Saprobni sekvencii pri semeystvo Simuliidae i znachenieyeto im za opredeliane kachestvo na vodata. Hydrobiology (Sofia) 9: 50–56.

Kovachev S (1979b) Bemerkungen über die Zusammensetzung, Verbreitung und Ökologie der Simuliiden (Diptera, Simuliidae) in der Bulgarischen Donauzuflüssen. XXI. Arbeitstagung IAD: 392–396.

Kovachev SG (1985a) Formiraneto na kachestveniya sŭstav na simulidnata fauna v reka Mesta - primer kŭm ideyata za saprobna sukcesiya. Hidrobiologiya 26: 49–52.

Kovachev SG (1985b) Simulidnijat kompleks ot reka Maritsa - primer za silno razrushena taksotsenoza. Hidrobiologija 26: 53–58.

Kovachev SG (2000) Qualitative Structure and Distribution of Black-Flies (Diptera, Simuliidae) from High Mountain Streams of Glacial Origin in the Rila Mountains. In: Golemansky V, Naidenov W (Eds) Biodiversity and Evolution of Glacial Water Ecosystems in the Rila Mountains. Institute of Zoology, Ministry of Environment and Waters, Sofia, 67-74.

Kovachev SG, Uzunov Y (1986) Formation of macroinvertebrate communities in the course of the biological selfpurification of the Mesta river. Archiv für Hydrobiologie Supplement 4: 427–526.

Kovachev S, Stoichev S, Uzunov Y, Kumanski K, Memeti A (1999) Hydrofaunistic study of the Tetovska River, Macedonia. Lauterbornia 36: 67–70.

Knoz J, Beuk PLTh (2020) Family Simuliidae. www. In: Beuk, P.L.Th. (Ed.) Checklist of the Diptera of the Netherlands. https://diptera-info.nl/news.php?fam=Simuliidae

Krčmárik S, López-Peña D, Kúdela M (in press) The first record of Simulium (Hellichiella) latipes (Meigen, 1804) in Slovakia (Diptera, Simuliidae) Check List 21:

Krek S, Kaćanski D, Tanasijević M (1976) Biocenološka analiza naselja insekata (Ephemeroptera, Plecoptera, Simuliidae i Psychodidae) sliva rijeke Sutjeske. Godishnjak Bioloshkog Instituta Univerziteta u Sarajevu 29: 23–54.

Kúdela M (2006) Die Verbreitung der Mitteleuropäischen Arten der Simulium variegatum Gruppe (Diptera: Simuliidae). Beiträge zur Entomologie Berlin 56(2): 465–75. <https://doi.org/10.21248/contrib.entomol.56.2.465-475>

Lock K (2018) Checklist of the Belgian blackflies (Diptera: Simuliidae). Bulletin de La Société Royale Belge d’Entomologie/ Bulletin van de Koninklijke Belgische Vereniging voor Entomologie 154: 228–236.

López-Peña D, Jiménez-Peydró R (2017) Updated checklist and distribution maps of blackflies (Diptera: Simuliidae) of Spain. The Simuliid Bulletin, 48: 14–45.

Makatov TK (2007) Vertikaľnoe rasprostranenie krovososushchikh moshek (Diptera, Simuliidae) v Pavlodarskom Priirtysh´e. Kazakhstanaa Zhene Shekaralas Aymaktaraa Ekologiyani Koreayn Zheane Tabigatty Payaalanyashch Ozekti Meseleleri 1: 352–354.

Makatov TK (2008a) Ekologo-faunisticheskiy obzor krovososushchikh moshek (Diptera, Simuliidae) vodotokov rek Irtysh i Shiderty Pavlodarskoy oblasti Kazakhstana. Vestnik Altayskogo Gosudarstvennogo Agrarnogo Universiteta 4(42): 28–32.

Makatov TK (2008b) Vidovoy sostav i ekologiya krovososushchikh moshek (Diptera, Simuliidae) severo-vostoka Kazakhstana. Vestnik Altayskogo Gosudarstvennogo Agrarnogo Universiteta 1(39): 38–42.

Mitrokhin VU (1973) Yaytsekladki moshek (Sem. Simuliidae) v Severnom Zauralie. Parazitologiya 7(1): 87–89.
