# Supplementary material for: European blackflies: accepted and questionable species (Diptera, Simuliidae)
Source: Zookeys. 2026 Sep 14;1291:251–81. doi: 10.3897/zookeys.1291.196803 (PMC13594990; doi:10.3897/zookeys.1291.196803)
Supplement: Supplementary file 2 [file zookeys-1291-251_article-196803__-s002.docx]

**Main article: Jedlička L, Kúdela M, Kúdelová T (2026) European black flies: accepted and questionable species (Diptera: Simuliidae).**

**Supplementary file 2. List of questionable blackfly species (Diptera: Simuliidae)**

Table S2 presents a list of questionable blackfly species whose presence in Europe has been reported but remains uncertain, either due to unclear species identity or identification or to doubts about the reliability of the records. The taxonomic system and nomenclature follow the Inventory (Adler 2025).

The table is vertically divided into four colour-coded sections:

**Green section:** Includes systematic categories from species to tribe, along with records of occurrence by country. This part is based on the most recent inventory (Adler 2025), with minor adjustments.

**Unfilled section:** Provides notes regarding the assessment of each species and its occurrence in Europe, including potential additions and sources that may have been omitted from the inventory. Categories: **NRR** – necessary record revision (necesse recordum revisionem), **OSL** – omit the species from the list (omittunt species ex list)

**Orange section:** Species names as used in Fauna Europaea (FaEu). Names are hyperlinked to the PESI database, which provides associated distributional information. “N/A” indicates the absence of an entry in FaEu.

**Blue section**: Shows the species name and distribution according to the *Catalogue of Palaearctic Diptera* (Rubtsov [Rubzov] & Yankovsky, 1988).

**Abbreviations used in the Catalogue (CAT) for species distribution:**

**Asia**: Refers to Asia excluding the former USSR, i.e. without the countries listed below under TC, KZ, SMA, WS, ES, and FE.

**Europe**: Denotes the part of Europe outside the former USSR, specifically including the area west of and including Finland, Poland, Slovakia, Romania, Bulgaria, and Greece.

**North Africa**: Includes Morocco, Algeria, Tunisia, Libya, Egypt, the Azores, Madeira, and the Canary Islands.

**USSR**:

**NET, CET, SET** – Northern, Central, and Southern European Territory (of the former USSR, now Russian Federation), respectively

**By** – Belarus

**Est** – Estonia

**La** – Latvia

**Li** – Lithuania

**Mo** – Moldova

**Rs** – Russia

**Uk** – Ukraine

**TC (Transcaucasia)**: **Arm** – Armenia, **Ay** – Azerbaijan, **Ge** – Georgia

**KZ** – Kazakhstan

**SMA (Soviet Middle Asia)**: **Ki** – Kyrgyzstan, **Ta** – Tajikistan, **Tu** – Turkmenistan, **Uz** – Uzbekistan

**WS, ES, FE**: Western Siberia, Eastern Siberia, and the (Russian) Far East, respectively.

For ease of use, the supplement is formatted without top or bottom margins; scrolling is recommended for viewing.

**Table S2.** List of questionable blackfly species

| INVentory (Adler 2025) | | | | | |  | present paper |  | FaEu/PESI | |  | CATaloque | |
| --- | --- | --- | --- | --- | --- | --- | --- | --- | --- | --- | --- | --- | --- |
| tribe | genus | subgenus | spp group | species | distribution according/in INV 2025 |  | **Category (bold, in black**)  addenda (in black) and notes (in blue) |  | name used | distribution |  | name used | distribution |
| Prosimuliini | | | | |  |  |  |  |  |  |  |  |  |
|  | *Helodon* | | | |  |  |  |  |  |  |  |  |  |
|  |  | *Helodon* | | |  |  |  |  |  |  |  |  |  |
|  |  |  |  | ***rubicundus*** Rubtsov, 1956 | Siberia (Irkutskaya ES, FE); Mongolia |  | **NRR**  Russia (NR): Bel’tyukova & Shubina (1977), Shubina (1986)  Russia (CR): Bel’tyukova (1962),  Siberia (WS): Aibulatov (2015) |  | N/A | N/A |  | *Helodon rubicundus* Rubtsov, 1956 | USSR: ES, FE |
|  | *Prosimulium* | | |  |  |  |  |  |  |  |  |  |  |
|  |  |  | *hirtipes* | |  |  |  |  |  |  |  |  |  |
|  |  |  |  | ***tridentatum*** Rubtsov, 1940 | Siberia (Irkutskaya ES, FE); Mongolia |  | **NRR**  Russia (KM): Aibulatov (2015),  Russia (NR): Shubina (1986) ex Ostroushko & Bel’tyukova (1983)  Siberia (WS): Petrozhitskaya & Rodkina (2009, Altai) |  | N/A | N/A |  | *Prosimulium tridentatum* Rubtsov, 1940 | USSR: ES, FE (Kamchatka) |
|  |  |  | *macropyga* | |  |  |  |  |  |  |  |  |  |
|  |  |  |  | ***ventosum*** Rubtsov, 1956 | Siberia (Irkutskaya ES) |  | **OSL**  Russia (NR): Ostroushko et al. (2007) ex Ostroushko & Bel’tyukova (1983) [only larvae] |  | N/A | N/A |  | *Prosimulium macropygum ventosum* Rubtsov, 1956 | USSR: ES |
| **Simuliini** | | | | |  |  |  |  |  |  |  |  |  |
|  | ***Metacnephia*** | | |  |  |  |  |  |  |  |  |  |  |
|  |  |  |  | ***persica*** (Rubtsov, 1940) | Iran; Armenia, Azerbaijan, Kazakhstan, Lebanon, Romania, Turkmenistan, Uzbekistan |  | **OSL**  dubious record from Romania |  | N/A | N/A |  | *Metacnephia persica* (Rubtsov, 1940)  incl. *M. p. hirta* (Rubtsov et Terterian, 1954) | USSR: KZ, SMA; Asia: Iran  Europe: R; USSR: TC |
|  |  |  |  | ***ramificata*** (Rubtsov, 1956) | Siberia (Khabarovsk FE); Mongolia, Romania, Uzbekistan |  | **OSL**  dubious record from Romania |  | [*Metacnephia ramificata* (Rubtsov, 1956)](https://www.eu-nomen.eu/portal/taxon.php?GUID=urn:lsid:faunaeur.org:taxname:219374) | East Palaearctic, Romania |  | *Metacnephia ramificata* (Rubtsov, 1956) | Europe: ?R; USSR: FE; Asia: Mongolia. |
|  |  |  |  | ***sommermanae*** (Stone, 1952) | USA (Alaska); Canada (Yukon), Kazakhstan, Siberia (ES, FE, WS), Uzbekistan, Romania (?) |  | **OSL**  dubious record from Romania |  | N/A | N/A |  | *Metacnephia* *crassifistula* (Rubtsov, 1956) | Europe: ?R; USSR: WS, ES, FE |
|  | ***Simulium*** | | |  |  |  |  |  |  |  |  |  |  |
|  |  | ***Eusimulium*** | | |  |  |  |  |  |  |  |  |  |
|  |  |  |  | ***brachyantherum*** Rubtsov, 1947 | Tajikistan; Armenia, Iran, Romania, Uzbekistan |  | **OSL**  dubious record from Romania |  | [*Simulium (Eusimulium) brachyantherum* Rubtsov, 1947](https://www.eu-nomen.eu/portal/taxon.php?GUID=urn:lsid:faunaeur.org:taxname:219413) | East Palaearctic, Bulgaria doubtfully present, Romania doubtfully present |  | *Eusimulium brachyantherum* (Rubtsov, 1947) | ?BG, R; USSR: TC, SMA. |
|  |  | ***Nevermannia*** | | |  |  |  |  |  |  |  |  |  |
|  |  |  | ***ruficorne*** | |  |  |  |  |  |  |  |  |  |
|  |  |  |  | ***ruficorne*** Macquart, 1838 | Malta, Portugal, Spain (only European records not covered in Cherairia and Adler (2018)) |  | **NRR**  taxonomic revision needed |  | [*Simulium (Nevermannia) ruficorne* Macquart, 1838](https://www.eu-nomen.eu/portal/taxon.php?GUID=urn:lsid:faunaeur.org:taxname:219486) | Afro-tropical region, East Palaearctic, Malta, North Africa, Portuguese mainland, Spanish mainland (incl. Balearic Isls in the map) |  | *Nevermannia ruficornis* (Macquart, 1838) | Europe: E, F; North Africa: Algeria, Morocco, Libya; Canary Is.; Afrotropical Region. |
|  |  |  | ***vernum*** | |  |  |  |  |  |  |  |  |  |
|  |  |  |  | ***fluviatile*** Radzivilovskaya, 1948 | Siberia (Maritime FE); China (Si), Romania (?) |  | **OSL**  dubious record from Romania |  | N/A | N/A |  | *Cnetha fluviatilis* (Radzivilovskaya, 1948) | Europe: ?R; USSR: FE. |
|  |  | ***Simulium*** | | |  |  |  |  |  |  |  |  |  |
|  |  |  | ***bezzii*** | |  |  |  |  |  |  |  |  |  |
|  |  |  |  | ***alajense*** Rubtsov, 1938 | Kyrgyzstan; Afghanistan, Armenia, Bulgaria, China (IM, Li, Tibet, Xi), India (Kashmir), Iran, Kazakhstan, Mongolia, Pakistan, Tajikistan, Turkey, Turkmenistan, Uzbekistan, Romania (?) |  | **NRR**  Bulgaria, Romania (?): based probably on Dinulescu’s (1965) record of *S. alajense hiemale* |  | [*Simulium (Simulium) alajense* Rubtsov, 1938](https://www.eu-nomen.eu/portal/taxon.php?GUID=urn:lsid:faunaeur.org:taxname:219520) | East Palaearctic, Bulgaria doubtfully present, Romania doubtfully present |  | *Tetisimulium alajense* Rubtsov,1938 | USSR: TC, KZ, SMA; Asia: Mongolia, China |
|  |  |  |  | ***desertorum*** Rubtsov, 1938 | China (Xinjiang, Jilin, Tibet); Bulgaria, Kazakhstan, Kyrgyzstan, Pakistan, Siberia (WS), Tajikistan, Turkmenistan |  | **NRR**  dubious record from Bulgaria |  | [*Simulium (Simulium) desertorum* Rubtsov, 1938](https://www.eu-nomen.eu/portal/taxon.php?GUID=urn:lsid:faunaeur.org:taxname:219541) | East Palaearctic, Bulgaria doubtfully present, |  | *Tetisimulium desertorum* (Rubtsov, 1939) | USSR: KZ, SMA; Asia: Northwestern China |
|  |  |  |  | ***hiemale*** (Rubtsov, 1956) | Azerbaijan; Armenia, Georgia, Uzbekistan. [‘China’ error] |  | **OSL**  Romania: Dinulescu (1965) as *S. alajense hiemale* |  | N/A | N/A |  | *Tetisimulium alajense hiemale* (Rubtsov, 1956) | Europe: BG, R; USSR: SET, TC. |
|  |  |  |  | ***kerisorum*** (Rubtsov, 1956) | Azerbaijan; Bulgaria, Kyrgyzstan, Turkey(?) |  | NRR  a single record from Bulgaria needs confirmation |  | [*Simulium (Simulium) kerisorum* (Rubtsov, 1956)](https://www.eu-nomen.eu/portal/taxon.php?GUID=urn:lsid:faunaeur.org:taxname:219555) | East Palaearctic, Bulgaria doubtfully present |  | *Tetisimulium kerisorum* (Rubtsov, 1956) | USSR: TC. |
|  |  |  |  | ***latimentum*** (Rubtsov, 1956) | Kyrgyzstan; Mongolia, Siberia (WS), Romania (?) |  | **OSL**  dubious record from Romania |  | [*Simulium (Simulium) latimentum* (Rubtsov, 1956)](https://www.eu-nomen.eu/portal/taxon.php?GUID=urn:lsid:faunaeur.org:taxname:219558) | Romania doubtfully present |  | *Tetisimulium latimentum* (Rubtsov, 1956) | Europe: ?R; USSR: SMA, WS |
|  |  |  | ***bimaculatum*** | |  |  |  |  |  |  |  |  |  |
|  |  |  |  | ***bimaculatum*** (Rubtsov, 1956) | Uzbekistan; Kazakhstan, China (?), Romania (?) |  | **OSL**  dubious record from Romania |  | [*Simulium (Simulium) bimaculatum* (Rubtsov, 1956)](https://www.eu-nomen.eu/portal/taxon.php?GUID=urn:lsid:faunaeur.org:taxname:219528) | East Palaearctic, Romania doubtfully present |  | *Odagmia bimaculata* Rubtsov, 1956: | Europe: ?R; USSR: KZ, SMA (Uz). |
|  |  |  | ***malyschevi*** | |  |  |  |  |  |  |  |  |  |
|  |  |  |  | ***malyschevi*** Dorogostaisky, Rubtsov & Vlasenko, 1935 | Siberia (Irkutskaya ES, FE); Canada (Alb, BC, Man, NWT, Sask, Yukon), China (He, Jilin, Li), Japan (Hon, Ky), Kazakhstan, Korea (South), USA (Alaska); |  | **OSL**  Mongolia: Yadamsuren et al. (2020)  Russia (NR): Bel’tyukova & Shubina (1977), Shubina (1986), Ostroushko et al. (2007), Sharkov et al. (1984) |  | N/A | N/A |  | *Gnus malyschevi* (Dorogostaisky, Rubtsov et Vlasenko, 1935)  *malyschevi albipes* Rubtsov, 1956  *malyschevi lucidum* Rubtsov, 1956 | USSR: ES, FE; Asia: China, ?Japan; USSR: ES, FE; USSR: ES |
|  |  |  | ***ornatum*** | |  |  |  |  |  |  |  |  |  |
|  |  |  |  | ***deserticola*** Rubtsov, 1940 | Tajikistan [neotype]; Mongolia, Romania, Ukraine, Uzbekistan |  | **OSL**  records from Romania and Ukraine are questionable |  | [*Simulium (Simulium) deserticola* Rubtsov, 1940](https://www.eu-nomen.eu/portal/taxon.php?GUID=urn:lsid:faunaeur.org:taxname:219540) | East Palaearctic, Romania doubtfully present |  | *Odagmia deserticola* (Rubtsov, 1940) | USSR: SMA; Asia: Mongolia |
|  |  |  |  | ***simoffi*** (Enderlein, 1924) | Bulgaria |  | **NRR**  nomen dubium |  | [*Simulium (Simulium) simoffi* (Enderlein, 1924)](https://www.eu-nomen.eu/portal/taxon.php?GUID=urn:lsid:faunaeur.org:taxname:219587) | Bulgaria |  | *Odagmia simoffi* Enderlein, 19ň24 | Europe: BG |
|  |  |  | ***pavlovskii*** | |  |  |  |  |  |  |  |  |  |
|  |  |  |  | ***pavlovskii*** Rubtsov, 1940 | Siberia (Maritime FE incl. Sakhalin, ES, Altai WS); China (He, IM, Xi), Mongolia, Ukraine (?) |  | **OSL**  Ukraine: according to Panchenko (2016) missing in Ukraine; the material used in Usovas's record (1987) of *pavlovskii* from the Ukrainian Carpathians was, according to a revision of the collection material (Sukhomlin & Zinchenko 2016), a misidentification of *murmanum* |  | N/A | N/A |  | *Gnus pavlovskii* (Rubtsov, 1940): | USSR: ES, FE; Asia: China |
|  |  |  | ***reptans*** | |  |  |  |  |  |  |  |  |  |
|  |  |  |  | ***kurense***  Rubtsov & Djafarov, 1951 | Azerbaijan; Armenia, Georgia, Uzbekistan |  | **OSL**  Bulgaria: Kovatschev (1976), not confirmed later |  | N/A | N/A |  | *Simulium (Simulium) kurense* Rubtsov et Djafarov, 1951 | USSR: TC |
|  |  |  |  | ***parvum*** Enderlein, 1921 | ‘Europe’ |  | **NRR**  species inquirenda |  | [*Simulium (Simulium) parvum* Enderlein, 1921](https://www.eu-nomen.eu/portal/taxon.php?GUID=urn:lsid:faunaeur.org:taxname:219571) | no record |  | *Simulium (Simulium) parvum* Enderelein, 1921 | Europe |
|  |  |  | ***variegatum*** | |  |  |  |  |  |  |  |  |  |
|  |  |  |  | ***exile***  (Rubtsov, 1956) | Azerbaijan; Kazakhstan, Romania |  | **NRR**  Russia (NR): Sharkov et al. (1984) ?  Romania: the record needs revision |  | [*Simulium (Simulium) exile* (Rubtsov, 1956)](https://www.eu-nomen.eu/portal/taxon.php?GUID=urn:lsid:faunaeur.org:taxname:219543) | East Palaearctic, Romania |  | *Odagmia exilis* Rubtsov, 1956 | Europe: R; USSR: TC. |
|  |  |  | ***venustum*** | |  |  |  |  |  |  |  |  |  |
|  |  |  |  | ***bergi***  Rubtsov, 1956 | Georgia; Armenia, Turkey, Ukraine |  | **NRR**  Ukraine: the records need revision |  | N/A | N/A |  | *Simulium (Simulium) bergi* Rubtsov, 1955 | USSR: TC |
|  |  |  |  | ***curvitarse* Rubtsov, 1940** | Siberia (Magadan FE, Kamchatka FE) China (He) |  | **NRR**  Russia (CR): Aibulatov 2013) - according to the known range the European records need revision |  | N/A | N/A |  | *Simulium (Simulium) curvitarse* Rubtsov, 1940 | USSR: FE; Asia: China |
|  |  | ***Wilhelmia*** | | |  |  |  |  |  |  |  |  |  |
|  |  |  | ***equinum*** | |  |  |  |  |  |  |  |  |  |
|  |  |  |  | ***turgaicum*** Rubtsov, 1940 | Kazakhstan; Afghanistan, Armenia, Azerbaijan, Bosnia and Herzegovina, China (IM, Xi), Iran, Iraq, Kyrgyzstan, Lebanon, Pakistan, Slovenia, Tajikistan, Turkey, Turkmenistan, Ukraine, Uzbekistan |  | **NRR**  Ukraine: missing, Sukhomlin & Zinchenko (2016) revising material stated that Usova’s (1987) record of the species from the steppe zone of Ukraine is based on misidentification (=*lineatum*).  Bosnia and Herzegovina and Slovenia: records are based on COI sequences which are not sufficient for identification (Duknič et al. 2019) |  | N/A | N/A |  | *Wilhelmia turgaica* (Rubtsov, 1940) | USSR: TC, KZ, SMA (Tu, Ki, Tj); Asia: Iran, China |
|  |  | **unplaced** | | |  |  |  |  |  |  |  |  |  |
|  |  |  |  | ***canescens*** Bremi, 1842 [*in* Kölliker] | Switzerland |  | **NRR**  species inquirenda |  | N/A | N/A |  | S*imulium canescens* Bréme, 1842 in Kölliker | not given, doubtful species |
|  |  |  |  | ***incanum*** Loew, 1840 | Poland |  | **NRR**  nomen dubium |  | N/A | N/A |  | *Simulium incanum* Loew, 1840 | Europe: PL |
|  |  |  |  | ***lividum*** (Schellenberg, 1803) | Switzerland |  | **OSL**  nomen dubium |  | N/A | N/A |  | N/A  *Hirtea livida* Schellenberg, 1803 mentioned but not listed as species | N/A |

**References**

(contains only references cited in this supplement)

Aibulatov SV (2013) Fauna moshek (Diptera: Simuliidae) Leningradskoy i Vologodskoy oblastey. Gidroentomogiya v Rossii Isopredeľnykh Stranakh: Materialy V Vserossiyskogo Simpoziuma po Amfibioticheskim i Vodnym Nasekomym 10–12.

Aibulatov SV (2015) A Contribution to the Fauna of Blackflies of the Subfamily Prosimuliinae (Diptera: Simuliidae) of the Sakha Republic (Yakutia). Entomological Review 95: 61–72. <https://doi.org/10.1134/S001387381501008X>

Bel’tyukova KN (1962) K izucheniju moshek (Simuliidae) v Permskoi oblasti. Uchenye Zapiski Permskogo Gosudarstvennogo Universiteta imeni A.M.Gorkogo 22(4): 91–100.

Bel’tyukova KN, Shubina VN (1977) Moshki (Diptera, Simuliidae) reki Shchugor i ikh roľ v pishche ryb. Biologicheskie Resursy Belogo Morya i Vnutrennikh Vodoemov Evropeyskogo Severa 1977: 38–40.

Cherairia M, Adler PH (2018) Genetic variation in a colonization specialist, *Simulium ruficorne* (Diptera: Simuliidae), the world’s most widely distributed black fly. PLOS one 13: e0205137.

Dinulescu, G. (1966). Insecta, Diptera, Fam. Simuliidae. (Muştele columbace). Fauna Republicii Socialiste Romȃnia 11 (8) Editura Academiei Republicii Socialiste Romȃnia. 600 pp.

Đuknić J, Jovanović VM, Popović N, Živić I, Raković M, Čerba D, Paunović M. (2019) Phylogeography of *Simulium* Subgenus *Wilhelmia* (Diptera: Simuliidae)—Insights from Balkan Populations. Journal of Medical Entomology 56(4): 967–978. <https://doi.org/10.1093/jme/tjz034>

Kovachev S (1976) Simuliidae (Diptera) ot porechiata na rekite Struma i Mesta. Hydrobiology (Sofia) 3: 67–77.

Ostroushko T S, Bel’tyukova KN (1983) Preimaginaľnye stadii moshek basseyna r. Shchugor. Trudy Komi Filiala AN SSSR 57: 68–77.

Ostroushko TS, Paniukova EV, Pestov CV (2007) Dvukrylye nasekomye (Insecta: Diptera) kompleksa ‘gnus’ fauny evropejskogo severo-vostoka Rossii. Trudy Komi Nauchnogo Centra UrO Rosiiskoi AN 183: 190–235. <https://doi.org/10.1134/S0013873807090114>

Panchenko AA (2016) Ob inventarizacii sovremennoy fauny moshek (Diptera: Simuliidae) materikovoy Ukrainy. X Verossiyskiy Dipterologicheskiy Simpozium, Krasnodar 261–266.

Petrozhitskaya, LV, Rodkina VI (2009) The Spatial Distribution of Black Flies (Diptera: Simuliidae) in the Basin of the Sema Mountain River in the North Altai Mountain Region. Inland Water Biology 2(1): 33–41. <https://doi.org/10.1134/S1995082909010064>

Sharkov AL, Lobkova ML, Usova ZV (1984) Krovososushchie Komary (Sem. Culicidae) i Moshki (Sem. Simuliidae) Evropeyskogo Severa SSSR. Petrozavodsk: Izd. Kareliya. 151 pp.

Shubina VN (1986) Gidrobiologiya lososevoy reki Severnogo Urala. Nauka, Leningrad, 158 pp.

Sukhomlin KB, Zinchenko OP (2016) Taksonomichna rizmanitist moshok (Diptera, Simuliidae). Ukrainskiy Entomologicheskiy Zhurnal, 11(1–2): 38–45.

Usova ZV (1987) Zonaľnoe raspredelenie moshek (Diptera, Simuliidae) Ukrainy. Krovososushchie Dvukrylye i ikh Kontroľ, 133–136.

Yadamsuren O, Morse JC, Hayford B, Gelhaus JK, Adler PH (2020) Macroinvertebrate community responses to land use: A trait-based approach for freshwater biomonitoring in Mongolia. Hydrobiologia, 847: 1887–1902. <https://doi.org/10.1007/s10750-020-04220-2>
